# Augmenting conventional criteria: a CT-based deep learning radiomics nomogram for early recurrence risk stratification in hepatocellular carcinoma after liver transplantation

**DOI:** 10.1186/s13244-025-02082-7

**Published:** 2025-09-17

**Authors:** Ziqian Wu, Danyang Liu, Siyu Ouyang, Jingyi Hu, Jie Ding, Qiu Guo, Jidong Gao, Jiawen Luo, Ke Ren

**Affiliations:** 1 Department of Radiology, Xiang’an Hospital of Xiamen University, School of Medicine, Xiamen University, Xiamen radiological Control Center, Xiamen, China; 2 Department of Radiology, The Second Affiliated Hospital of Dalian Medical University, Dalian, China; 3 Department of Radiology, The Third Affiliated Hospital of Sun Yat-Sen University, Guangzhou, China; 4 Department of Radiology, The First Affiliated Hospital of Xiamen University, School of Medicine, Xiamen University, Fujian, China

**Keywords:** Hepatocellular carcinoma, Liver transplantation, Early recurrence, Radiomics, Deep learning

## Abstract

**Background:**

We developed a deep learning radiomics nomogram (DLRN) using CT scans to improve clinical decision-making and risk stratification for early recurrence of hepatocellular carcinoma (HCC) after transplantation, which typically has a poor prognosis.

**Materials and methods:**

In this two-center study, 245 HCC patients who had contrast-enhanced CT before liver transplantation were split into a training set (*n* = 184) and a validation set (*n* = 61). We extracted radiomics and deep learning features from tumor and peritumor areas on preoperative CT images. The DLRN was created by combining these features with significant clinical variables using multivariate logistic regression. Its performance was validated against four traditional risk criteria to assess its additional value.

**Results:**

The DLRN model showed strong predictive accuracy for early HCC recurrence post-transplant, with AUCs of 0.884 and 0.829 in training and validation groups. High DLRN scores significantly increased relapse risk by 16.370 times (95% CI: 7.100–31.690; *p*  < 0.001). Combining DLRN with Metro-Ticket 2.0 criteria yielded the best prediction (AUC: training/validation: 0.936/0.863).

**Conclusion:**

The CT-based DLRN offers a non-invasive method for predicting early recurrence following liver transplantation in patients with HCC. Furthermore, it provides substantial additional predictive value with traditional prognostic scoring systems.

**Critical relevance statement:**

AI-driven predictive models utilizing preoperative CT imaging enable accurate identification of early HCC recurrence risk following liver transplantation, facilitating risk-stratified surveillance protocols and optimized post-transplant management.

**Key Points:**

- A CT-based DLRN for predicting early HCC recurrence post-transplant was developed.
- The DLRN predicted recurrence with high accuracy (AUC: 0.829) and 16.370-fold increased recurrence risk.
- Combining DLRN with Metro-Ticket 2.0 criteria achieved optimal prediction (AUC: 0.863).

**Graphical Abstract:**

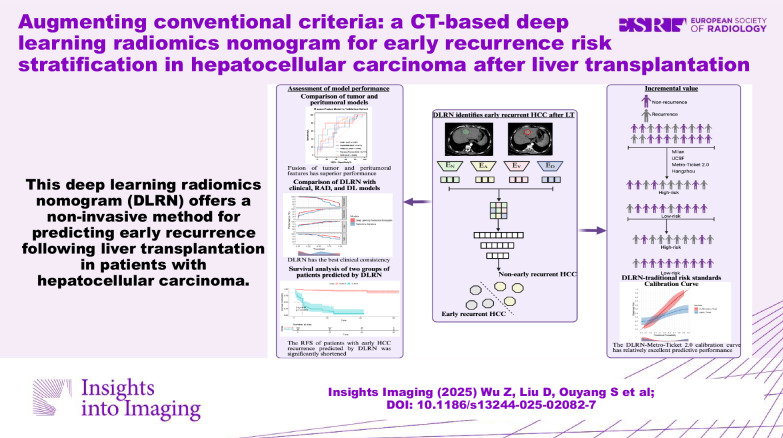

## Introduction

Hepatocellular carcinoma (HCC) ranks as the third leading cause of cancer-related mortality globally, with a relative 5-year survival rate of approximately 18% [1]. The close association between morbidity and mortality highlights the severe prognosis of this disease [2]. Liver transplantation (LT) currently represents the preferred treatment modality, offering substantial survival advantages to patients [3]. However, given the limited availability of donor organs, it is imperative that organ allocation policies optimize patient outcomes while minimizing ineffective therapeutic interventions [4]. Presently, the most commonly employed criteria include the Milan criteria; the University of California, San Francisco (UCSF) criteria (which consider tumor number and size); the Metro-Ticket 2.0 model (which integrates biological characteristics); and the Hangzhou criteria. Despite rigorous screening protocols, HCC recurs in 10–15% of patients following LT [5]. Notably, early recurrence of HCC (within 2 years post-transplantation) often exhibits an aggressive clinical trajectory and is associated with elevated mortality rates [6, 7].

At present, artificial intelligence (AI) is extensively utilized in the domain of medical imaging, primarily to enhance diagnostic accuracy and optimize decision-making processes. Radiomics is dedicated to the high-throughput extraction and analysis of quantitative imaging features from medical images, and it is regarded as an effective method for capturing the intrinsic heterogeneity of solid tumors [8]. In comparison to invasive tissue biomarkers, quantitative features derived from CT or MRI images have demonstrated substantial advancements in predicting tumor classification [9], grading [10], and prognosis [11, 12] in HCC. Deep learning (DL), a significant branch and emerging direction within AI, augments the capability to express complex tasks through neural networks, facilitating machine-automated feature extraction [13]. The residual convolutional network (ResNet) represents one of the deep convolutional neural network architectures. Its core innovation lies in the incorporation of “residual learning” and “skip connections,” which effectively address the prevalent issues of gradient vanishing and gradient exploding during the training of deep networks, thereby markedly enhancing network performance and training efficiency [14]. ResNet has demonstrated outstanding performance in predicting vascular encapsulation of tumor clusters (VETC) [15] and microvascular invasion (MVI) [16] in HCC. Nonetheless, there is a paucity of literature regarding DL radiomics studies on early recurrence following LT in HCC patients and their additional value to existing standards.

Initial research predominantly concentrated on the radiomics analysis of the internal tumor region. However, recent investigations have increasingly highlighted the significance of the tumor microenvironment in cancer progression, underscoring its substantial impact on tumor biological behavior and patient prognosis [17, 18]. Recent findings indicate that a model integrating radiomics features from both the tumor interior and the adjacent 5 mm region markedly enhances the predictive accuracy for MVI in patients with small HCC (tumor diameter ≤ 5 cm) [19]. Currently, there is insufficient evidence to ascertain whether the imaging characteristics of the tumor microenvironment surrounding HCC can reliably predict early recurrence in patients post-LT.

This study develops a CT-based deep learning radiomics nomogram (DLRN) to predict early recurrence in HCC patients post-LT and assesses its added value to current selection criteria.

## Materials and methods

### Patients

This study was conducted with the Declaration of Helsinki. Conducted retrospectively at two centers with Institutional Review Board approval (ethics approval no. XAHLL2024035), it included 245 out of 423 HCC patients from Xiamen University Xiang’an Hospital and the Second Affiliated Hospital of Dalian Medical University, spanning January 2019 to June 2023. The training cohort comprised 184 patients from Xiamen, while 61 patients from Dalian formed the validation cohort. Inclusion and exclusion criteria are detailed in Supplementary Information (E1.1) and Fig. S1. Detailed descriptions of the surgical methodologies employed in LT are comprehensively outlined in E1.2.

### Follow-up and study endpoints

All patients were monitored for a minimum duration of two years following LT, with the final follow-up occurring on June 1, 2025. During the first postoperative month, patients underwent screening for HCC recurrence through the assessment of serum alpha-fetoprotein (AFP) levels, ultrasound examinations, and enhanced CT or MRI of the chest and abdomen. Screening was conducted quarterly during the first year and biannually thereafter.

The primary endpoint of the study was early recurrence, defined as the occurrence of any of the following within two years post-LT: (i) the emergence of new intrahepatic lesions exhibiting typical HCC imaging characteristics; (ii) HCC confirmed via biopsy, postoperative pathology, or tumor staining following postoperative transarterial chemoembolization (TACE), despite atypical imaging presentations; and (iii) extrahepatic metastasis confirmed through characteristic imaging features or histological analysis.

### CT protocols

CT images were obtained in the transverse axial plane, encompassing the noncontrast phase (N), arterial phase (A), portal venous phase (V), and delayed phase (D). Detailed imaging protocols are provided in the Supplementary Information (E1.3), and the CT scan parameters are listed in Table S1.

### Clinical features and radiological evaluation

The study encompasses several key components: (i) Demographic data, which include age, sex, time to early relapse, hepatitis virus infection status, liver cirrhosis, and the Barcelona Clinic Liver Cancer (BCLC) stage. (ii) Clinical and laboratory characteristics, such as AFP, carcinoembryonic antigen (CEA), alanine aminotransferase (ALT), aspartate aminotransferase (AST), model for end-stage liver disease (MELD) score, Child–Pugh grade, Ki-67 proliferation index, cytokeratin-19 (CK-19), MVI (defined as the presence of cancer cell nests within the lumen of endothelial cells under microscopy [20]), histological grade (categorized as well-differentiated, moderately differentiated, or poorly differentiated), and bridging therapy prior to transplantation. Additionally, the study evaluates whether patients met the Milan, UCSF, Metro-Ticket 2.0, and Hangzhou criteria [21].

In cases involving multiple HCCs, the largest tumor was designated as the primary subject of analysis. Two radiologists (J.D. and J.H., possessing 15 years and 5 years of experience in abdominal imaging, respectively) independently and blindly reviewed the imaging films to assess the features. In instances of disagreement, a consensus was achieved through consultation. The radiological assessment focused on five features: (i) the number of tumors; (ii) maximum tumor diameter; (iii) tumor margin; (iv) peritumoral enhancement during the arterial phase; and (v) the presence of a pseudocapsule (E1.4 and Fig. S2).

### Radiomics and DL feature extraction

**Fig. 1:**
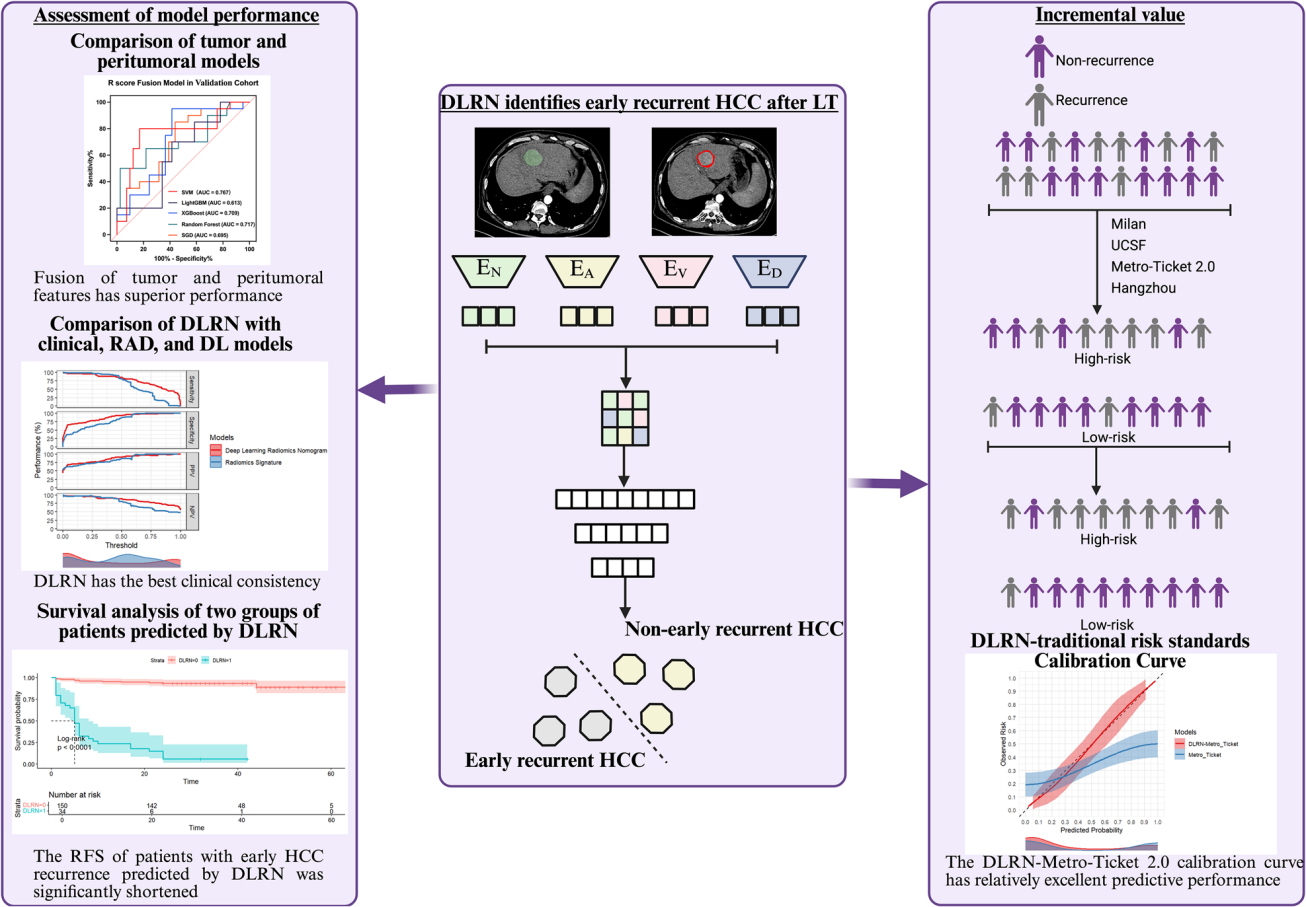
The workflow of the study

The workflow is depicted in Fig. 1. Prior to segmentation, the original CT images underwent registration to produce the registered images (E1.5). Reader 1 (J.H.) manually delineated the tumor on the transverse axial plane of the portal venous phase images utilizing 3D-Slicer (version 5.2.2, https://www.slicer.org/). Subsequently, a 5-mm-wide band was automatically generated by expanding the tumor boundary, and the non-liver regions were manually excised through a meticulous review of each image. The volume of interest (VOI) used for radiomics and DL feature extraction covers the tumor and peritumoral areas. To assess feature stability, reader 1 (J.H.) performed the segmentation twice within a two-week interval, while reader 2 (J.D.) independently delineated the segmentation area in a cohort of 30 randomly selected patients. The intraclass correlation coefficients (ICC) for both inter- and intra-observer reliability were ≥ 0.75.

Following image preprocessing, radiomic features were extracted from the previously defined four-phase VOIs using the PyRadiomics package. Additionally, DL features were extracted from the four-phase VOIs using a pre-trained ResNet-18 model [14]. Further details are available in the Supplementary Materials (E1.6 and E1.7), Table S2, and Fig S3.

### Feature selection and model building

Feature selection and model construction were exclusively conducted on the training dataset. The feature selection process involved the application of the Mann–Whitney *U*-test, Spearman correlation analysis, and the least absolute shrinkage and selection operator (LASSO) algorithm (refer to E1.8 for further details). Since the dataset was imbalanced in the training dataset, a synthetic minority over-sampling technique (SMOTE) was used to resample the training dataset by applying a “minority” resampling strategy. After sample resampling, the ratio between early recurrence cases and non-early recurrence cases was close to 1:1.

Separate models for tumor and peritumoral regions were developed based on the radiomic features of the respective VOI. To assess whether the integration of tumor and peritumoral data could enhance diagnostic accuracy, a feature-level fusion was executed. This involved merging the radiomic features from both regions into a single, comprehensive feature set, followed by feature selection and score construction. For the development of the radiomic score (RAD-score), five distinct machine learning classifiers—support vector machine (SVM), lightweight gradient boosting decision tree (LightGBM), extreme gradient boosting (XGBoost), random forest, and stochastic gradient descent (SGD)—were evaluated within the training cohort using 10-fold cross-validation. The aforementioned selected features served as input variables for these classifiers. Ultimately, the model demonstrating the highest area under the curve (AUC) in the validation cohort was selected for final construction. The DL-score was derived by integrating the selected features with their respective weights. Independent clinical variables identified through the logistic regression model were amalgamated with the RAD-score and DL-score to develop a DLRN. Collinearity analysis was performed on clinical factors after univariate analysis with RAD and DL characteristics. The evaluation factors were tolerance and variance inflation factor (VIF); tolerance values < 0.1 or VIF values > 5 were considered to indicate collinearity between two variables. Additionally, multivariate logistic regression analysis was conducted using solely independent clinicopathological variables to construct clinical models for comparative purposes. All models underwent validation within the validation cohort and were quantitatively compared.

The radiomics quality score was computed to assess the methodologies, analyses, and reporting of our study (https://www.radiomics.world/rqs2).

### The incremental value of DLRN to existing risk standards

Within the entire cohort, the Milan, UCSF, Metro-Ticket 2.0, and Hangzhou criteria were validated for their predictive capability regarding recurrence-free survival (RFS) following LT in patients with HCC. Subsequently, Kaplan–Meier analysis was employed to assess the efficacy of the dichotomous DLRN score in further stratifying patient subgroups both within and outside the four traditional criteria. Finally, four composite nomograms (i.e., D-Milan, D-UCSF, D-Metro-Ticket 2.0, and D-Hangzhou) were constructed to evaluate the supplementary value of DLRN in relation to the existing four risk criteria.

### Statistical analyses

Continuous variables were characterized by medians and interquartile ranges (IQRs), whereas categorical variables were expressed as frequencies and percentages. To identify pertinent risk factors, univariate and multivariate logistic regression analyses were conducted to compute odds ratios (ORs) along with their 95% confidence intervals (CIs). The DeLong test was employed to compare the performance of various models. The models’ ability to discriminate early recurrence of HCC following LT was assessed using the AUC. Calibration plots were utilized to compare the consistency between predicted values and actual observations, and the Brier score was used to evaluate the models’ goodness of fit. A *p*-value of less than 0.05 was considered indicative of statistically significant differences. All statistical analyses were executed using Python (version 3.9.6, https://www.python.org/) and R software (version 4.2.0, http://www.Rproject.org).

## Results

### Clinical and CT radiological characteristics

The clinical parameters of the patients are detailed in Table 1, which indicates no significant differences in the baseline characteristics between the two groups. The median follow-up duration was 29 months, with a range of 24–66 months. As of the most recent follow-up, the early recurrence rate in the training cohort was 23.91% (44 out of 184), while the validation cohort exhibited an early recurrence rate of 32.79% (20 out of 61), with no statistically significant difference between the two groups (*p* = 0.231). The median RFS for patients experiencing early recurrence was 5 months, with a range of 1–24 months.

**Table 1:** Demographic, clinical, and tumor characteristics of the LT population

Characteristics	Total (*n* = 245)	Training set (*n* = 184)	Validation set (*n* = 61)	*p*-value
Patient demographics				
Age (years)	53.0 (46.0–58.0)	53.0 (46.0–56.0)	52.0 (48.0–60.0)	0.330
Gender				0.063
Male	202 (82.5)	157 (85.3)	45 (73.8)	
Female	43 (17.6)	27 (14.7)	16 (26.2)	
Etiology				0.205
HBV	215 (87.8)	161 (87.5)	54 (88.5)	
HCV	6 (2.5)	3 (1.6)	3 (4.9)	
Others	24 (9.8)	20 (10.9)	4 (6.6)	
Liver cirrhosis				0.769
Absent	37 (15.1)	29 (15.8)	8 (13.1)	
Present	208 (84.9)	155 (84.2)	53 (86.9)	
BCLC stage				0.178
0 + A	130 (53.1)	105 (57.1%)	25 (41.0%)	
B	70 (28.6)	49 (26.6%)	21 (34.4%)	
C	27 (11.0)	18 (9.8%)	9 (14.8%)	
D	18 (7.4)	12 (6.5%)	6 (9.8%)	
Clinical parameters				
AFP (ng/mL)	16.6 (3.1–282.0)	8.9 (3.1–235.0)	51.9 (3.2–334.9)	0.288
ALT (U/L)	29.0 (19.0–43.4)	27.7 (18.2–44.3)	35.6 (27.7–43.3)	0.103
AST (U/L)	43.9 (30.7–70.0)	41.2 (29.0–62.0)	49.7 (36.0–80.2)	0.107
CEA (ng/mL)	2.19 (1.4–4.1)	2.2 (1.4–3.8)	2.16 (1.4–4.3)	0.841
MELD score	10.0 (6.0–15.0)	9.0 (6.0–14.0)	10.0 (7.0–17.0)	0.121
Child–Pugh				0.178
A	130 (53.1)	98 (53.3)	32 (52.5)	
B	102 (41.6)	79 (42.9)	23 (37.7)	
C	13 (5.3)	7 (3.8)	6 (9.8)	
Ki-67 PI				0.410
Ki-67 ≤ 10%	47 (19.2)	38 (20.7)	9 (14.8)	
Ki-67 > 10%	198 (80.8)	146 (79.4)	52 (85.3)	
CK-19				0.980
Absent	167 (68.2)	126 (68.5)	41 (67.2)	
Present	78 (31.8)	58 (31.5)	20 (32.8)	
MVI				0.071
Absent	158 (64.5)	125 (67.9)	33 (54.1)	
Present	87 (35.5)	59 (32.1)	28 (45.9)	
Pre-LT treatment				0.190
Absent	74 (30.2)	51 (27.7)	23 (37.7)	
Present	171 (69.8)	133 (72.3)	38 (62.3)	
Histologic grade				0.255
Well differentiated	56 (22.9)	43 (23.4)	13 (21.3)	
Moderately differentiated	141 (57.6)	101 (54.9)	40 (65.6)	
Poorly differentiated	48 (19.6)	40 (21.7)	8 (13.1)	
Milan criteria				0.741
Within	139 (56.7)	106 (57.6)	33 (54.1)	
Beyond	106 (43.3)	78 (42.4)	28 (45.9)	
UCSF criteria				1.000
Within	179 (73.1)	134 (72.8)	45 (73.8)	
Beyond	66 (26.9)	50 (27.2)	16 (26.2)	
Metro-Ticket2.0 model				0.358
Within	174 (71.0)	134 (72.8)	40 (65.6)	
Beyond	71 (29.0)	50 (27.2)	21 (34.4)	
Hangzhou criteria				0.101
Within	217 (88.6)	167 (90.8)	50 (82.0)	
Beyond	28 (11.4)	17 (9.2)	11 (18.0)	
Radiologic features				
Tumor number				0.329
Single	87 (35.5)	69 (37.5)	18 (29.5)	
Multiple	158 (64.5)	115 (62.5)	43 (70.5)	
Maximum tumor diameter (cm)	3.5 (2.0–6.5)	3.5 (2.0–6.7)	4.0 (2.5–6.5)	0.361
Tumor borderline, clear				0.910
Absent	116 (47.4)	88 (47.8)	28 (45.9)	
Present	129 (52.7)	89 (52.2)	23 (54.1)	
AP peritumoral enhancement				0.096
Absent	128 (52.2)	90 (48.9)	38 (63.2)	
Present	117 (47.8)	94 (51.1)	23 (37.7)	
Pseudocapsule, well-defined				0.320
Absent	55 (22.5)	38 (20.7)	17 (27.9)	
present	190 (77.6)	146 (79.4)	25 (72.1)	

Data are presented as numbers (%) or medians (interquartile range, IQR)

*HBV* hepatitis B virus, *HCV* hepatitis C virus, *AFP* alpha-fetoprotein, *ALT* alanine aminotransferase, *AST* aspartate aminotransferase, *CEA* carcinoembryonic antigen, *BCLC* Barcelona Clinic Liver Cancer, *PI* proliferation index, *CK* cytokine, *AP* arterial phase, *MVI* microvascular invasion, *LT* liver transplantation, *Pre-LT* treatment strategies included surgery, ablation, transarterial chemoembolization, chemotherapy, and targeting therapy, *UCSF* University of California San Francisco

Univariate analysis of the training cohort identified several variables significantly associated with early recurrence of HCC following LT, including age, BCLC stage, Ki-67 proliferation index, MVI, histological grade, maximum tumor diameter, presence of a pseudocapsule, and peritumoral enhancement during the arterial phase (all *p* < 0.05). Multivariate analysis further revealed that BCLC stage (OR = 9.30; 95% CI: 3.00–58.10; *p* = 0.019), MVI (OR = 3.96; 95% CI: 1.53–10.24; *p* = 0.005), and peritumoral enhancement during the arterial phase (OR = 2.86; 95% CI: 1.14–7.21; *p* = 0.026) were independently associated with early recurrence (Tables S3 and S4).

### Feature selection and model building validation

**Fig. 2:**
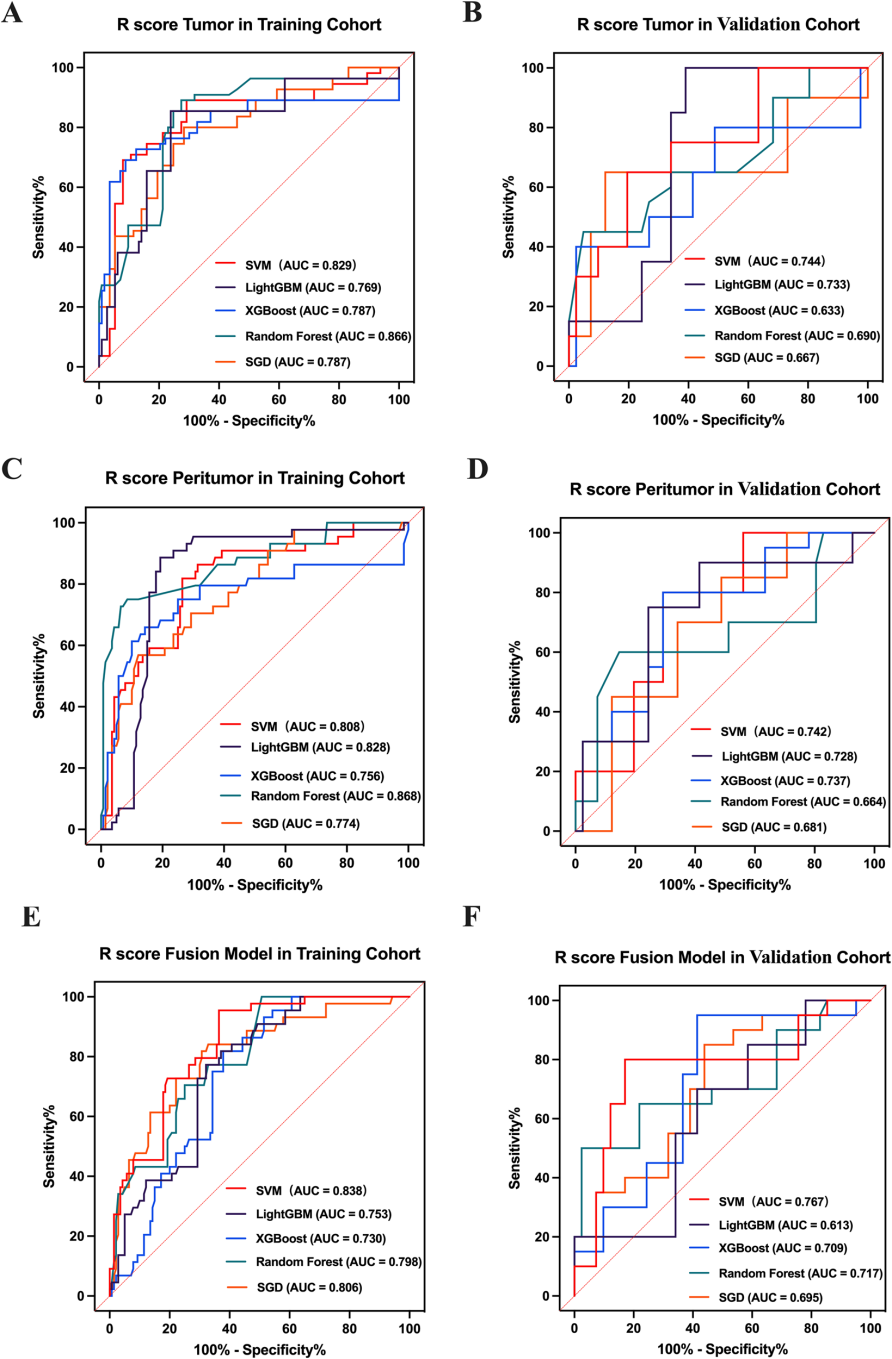
Receiver operating characteristic (ROC) curves for various models predicting early recurrence following LT for HCC are presented for both training sets (**A**, **C**, **E**) and validation sets (**B**, **D**, **F**). The fusion model incorporates features from both the tumor and its peritumoral areas. AUC, area under the curve; SVM, support vector machine; LightGBM, light gradient boosting machine; XGBoost, extreme gradient boosting; SGD, stochastic gradient descent

Following feature selection, the radiomics model development retained 13 features from the tumor VOI, 6 features from the peritumoral VOI, and 19 features from the combination of tumor and peritumoral characteristics (Table S5). The performance of the five classifiers is depicted in Fig. 2 and Table S6. The random forest classifier exhibited an overfitting issue, demonstrating strong performance in the training cohort but suboptimal performance in the validation cohort, with training/validation AUC values of 0.866/0.690, 0.868/0.664, and 0.798/0.717, respectively. In contrast, the SVM outperformed the other classifiers in the validation cohort, achieving AUCs of 0.744 (95% CI: 0.613–0.875), 0.742 (95% CI: 0.617–0.866), and 0.767 (95% CI: 0.626–0.908). The SVM classifier emerged as the most effective classifier within the fusion model, which integrates both tumor and peritumoral radiomic features, and was consequently utilized for further analysis. The RAD-score was derived from this radiomics model. For the development of the DL model, 11 features were selected. The formulas for calculating the RAD-score and DL-score are provided in the supplementary material (E1.9). The tolerance range of the variables was 0.623–0.905, and the VIF range was 1.120–1.604, indicating that there was no collinearity between the variables (Table S7).

**Fig. 3:**
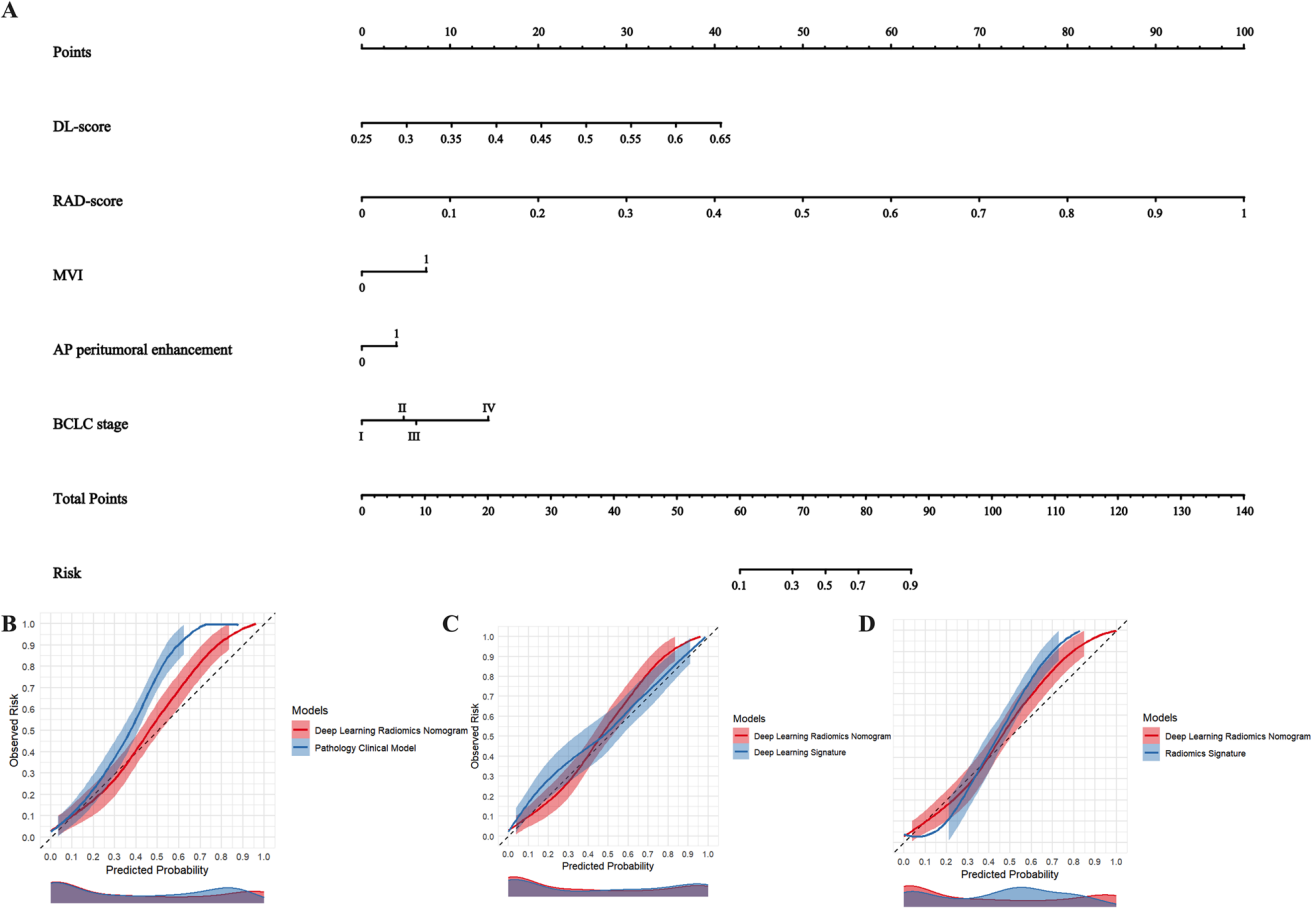
DLRN and calibration curve. **A** The deep learning radiomics nomogram (DLRN) for predicting early recurrence of hepatocellular carcinoma after liver transplantation. **B**–**D** Show the calibration curves of DLRN and pathological clinical model (**B**), deep learning model (**C**), and radiomics model (**D**). DL-score, deep learning score; RAD-score, radiomic score; MVI, microvascular invasion; AP, arterial phase; BCLC, Barcelona Clinic Liver Cancer

The DLRN model was developed by integrating the BCLC stage, MVI, AP peritumoral enhancement, RAD-score, and DL-score (Fig. 3A). Calibration curves for the pathological clinical model, DL model, RAD model, and DLRN are depicted in Fig. 3B–D, demonstrating that the survival rate predictions by DLRN closely aligned with the actual outcomes. All groups exhibited Brier scores below 0.250, with DLRN achieving a score of 0.166, the pathological clinical model 0.209, the DL model 0.188, and the RAD model 0.182. Decision curve analysis indicated that DLRN offered a greater net benefit within a reasonable threshold probability range compared to the other three models (Fig. S4A). The performance metrics of the pathological clinical model, DL model, RAD model, and DLRN are detailed in Table 2 and Fig. S4B–D. Notably, the AUC for DLRN in the validation cohort was 0.829 (95% CI: 0.789–0.868). Overall, DLRN demonstrated quantitative superiority over other predictive models.

**Table 2:** The performance of different signature models

Datasets	Model	Sensitivity (95% CI)	Specificity (95% CI)	Accuracy (95% CI)	AUC (95% CI)
Training set	DLRN	0.935 (0.834–0.976)	0.814 (0.739–0.874)	0.887 (0.833–0.927)	0.884 (0.851–0.917)
	DL	0.887 (0.771–0.952)	0.813 (0.738–0.873)	0.792 (0.728–0.847)	0.812 (0.780–0.844)
	RAD	0.927 (0.822–0.975)	0.810 (0.735–0.870)	0.887 (0.833–0.927)	0.856 (0.822–0.890)
	Pathology clinical	0.761 (0.619–0.865)	0.733 (0.654–0.801)	0.756 (0.689–0.814)	0.751 (0.728–0.775)
Validation set	DLRN	0.877 (0.783–0.952)	0.870 (0.817–0.923)	0.875 (0.792–0.918)	0.829 (0.789–0.868)
	DL	0.767 (0.682–0.852)	0.686 (0.644–0.728)	0.750 (0.641–0.859)	0.739 (0.713–0.764)
	RAD	0.770 (0.686–0.854)	0.793 (0.719–0.867)	0.774 (0.719–0.829)	0.793 (0.762–0.824)
	Pathology clinical	0.720 (0.523–0.817)	0.615 (0.516–0.714)	0.684 (0.617–0.751)	0.670 (0.549–0.790)

Models are named after the feature type

*DLRN* deep learning radiomics nomogram, *DL* deep learning, *RAD* radiomic, *AUC* area under the receiver operating characteristic curve, *CI* confidence interval.

### Association between DLRN and survival outcomes

**Fig. 4:**
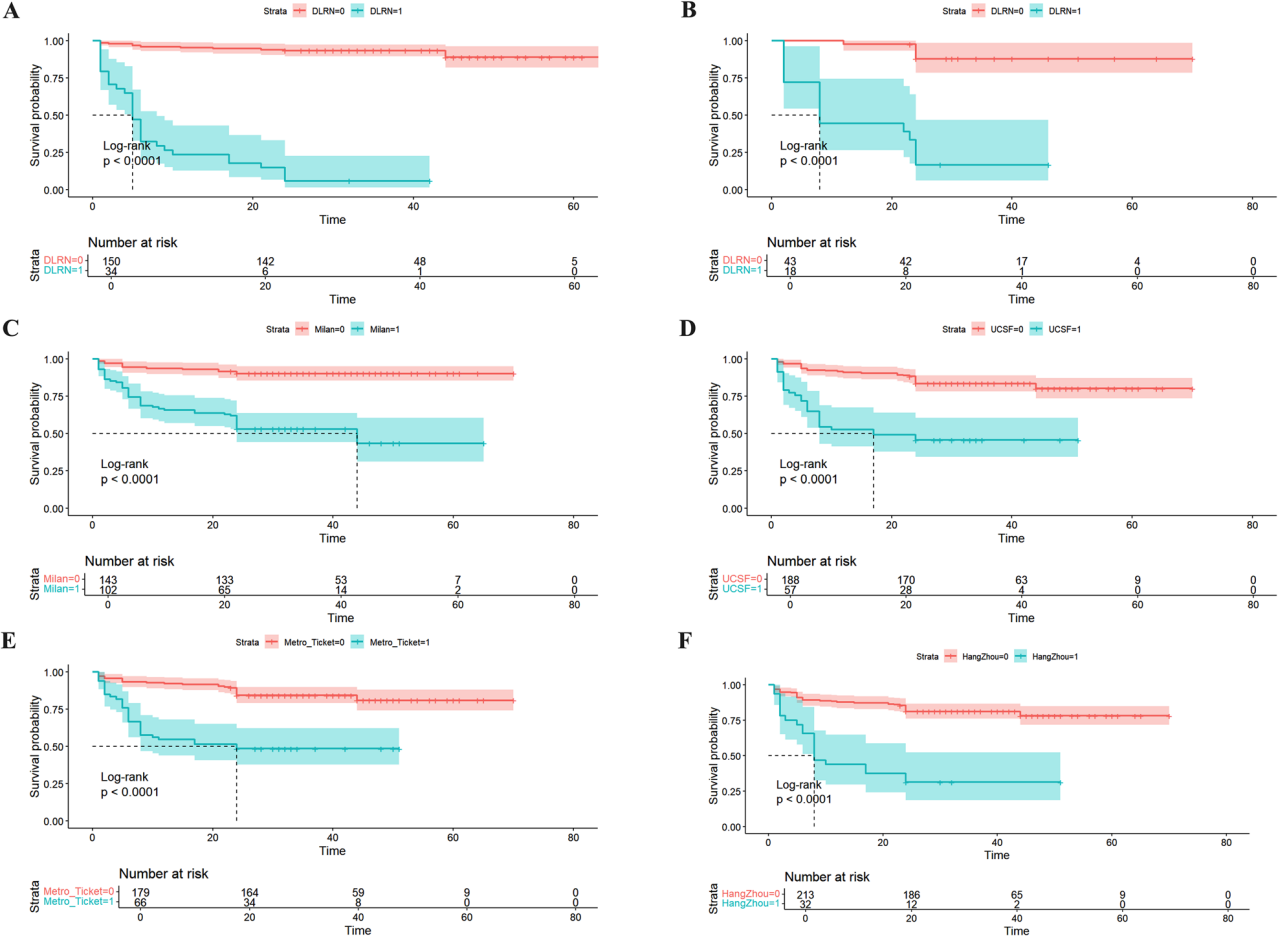
Kaplan–Meier survival curves of RFS in the entire cohort using the DLRN score in the training cohort (**A**) and the validation cohort (**B**), and the Milan criteria (**C**), UCSF (**D**), Metro-Ticket 2.0 (**E**), and Hangzhou (**F**) criteria. Strata = 0, low DLRN score group or within the traditional risk criteria; Strata = 1, high DLRN score group or outside the traditional risk criteria. UCSF, University of California, San Francisco

We have validated the prediction model for early recurrence of HCC post-LT in the validation cohort, confirming the model’s robustness and accuracy. Furthermore, the predictive assessment of survival outcomes remains a critical consideration. In this study, we conducted a comparative analysis between the DLRN-based prediction model and conventional classification criteria for predicting survival outcomes. The optimal cutoff value for the DLRN score was determined to be 0.724, which facilitated the stratification of patients into high and low DLRN score groups. Kaplan–Meier survival analysis revealed a significant correlation between the DLRN score and RFS in both the training and validation cohorts (log-rank *p*  < 0.001). Specifically, patients with low DLRN scores exhibited significantly longer RFS compared to those with high DLRN scores (Fig. 4A, B). The risk of relapse was 16.370 times higher in patients with high DLRN scores than in those with low scores (95% CI: 7.100–31.690; *p*  < 0.001). Figure 4C–F illustrates the Kaplan–Meier survival curves for the Milan, UCSF, Metro-Ticket 2.0, and Hangzhou criteria across the entire cohort. Patients who did not meet these four traditional criteria experienced shorter median RFS and faced a more severe prognosis. Table 3 presents the estimated hazard ratios (HR) for early HCC recurrence post-LT in patients classified within and outside the traditional criteria.

**Table 3:** Estimated HRs for early recurrence of HCC after LT in different risk groups and subgroups stratified by Milan, UCSF, Metro-Ticket 2.0, and Hangzhou criteria

Patients	Group/subgroup	HR (95% CI)	*p*-value
All patients	Beyond Milan vs within Milan	3.384 (1.671–5.195)	< 0.001
All patients	Beyond UCSF vs within UCSF	4.162 (2.536–6.832)	< 0.001
All patients	Beyond Metro-Ticket 2.0 vs within Metro-Ticket 2.0	4.091 (2.019–7.019)	< 0.001
All patients	Beyond Hangzhou vs within Hangzhou	4.333 (2.493–6.482)	< 0.001
Within Milan patients	High DLRN-score vs low DLRN-score	12.520 (6.025–28.248)	< 0.001
Beyond Milan patients	High DLRN-score vs low DLRN-score	13.944 (6.559–34.714)	< 0.001
Within UCSF patients	High DLRN-score vs low DLRN-score	13.227 (7.009–26.877)	< 0.001
Beyond UCSF patients	High DLRN-score vs low DLRN-score	14.910 (6.051–28.938)	< 0.001
Within Metro-Ticket 2.0 patients	High DLRN-score vs low DLRN-score	12.731 (6.568–22.702)	< 0.001
Beyond Metro-Ticket 2.0 patients	High DLRN-score vs low DLRN-score	14.640 (7.361–31.924)	< 0.001
Within Hangzhou patients	High DLRN-score vs low DLRN-score	11.900 (5.991–20.751)	< 0.001
Beyond Hangzhou patients	High DLRN-score vs low DLRN-score	10.042 (4.880–23.348)	< 0.001

*CI* confidence intervals, *UCSF* University of California San Francisco, *DLRN-score* Deep learning radiomics nomogram score

### The incremental value of DLRN to traditional risk criteria

In addition, Kaplan–Meier survival curves pertaining to early recurrence were constructed based on DLRN scores within subgroups stratified by the Milan, UCSF, Metro-Ticket 2.0, and Hangzhou criteria (Fig. S5). The analysis revealed a significant association between the DLRN score and early recurrence, both within and beyond the patient populations defined by these four traditional risk criteria (*p* < 0.001). Subgroup analysis demonstrated that the HR value was substantially higher compared to the traditional risk criteria alone. Specifically, the risk of early HCC recurrence following LT in patients with elevated DLRN scores was 10.042–14.910 times greater than in those with lower DLRN scores (Table 3).

**Fig. 5:**
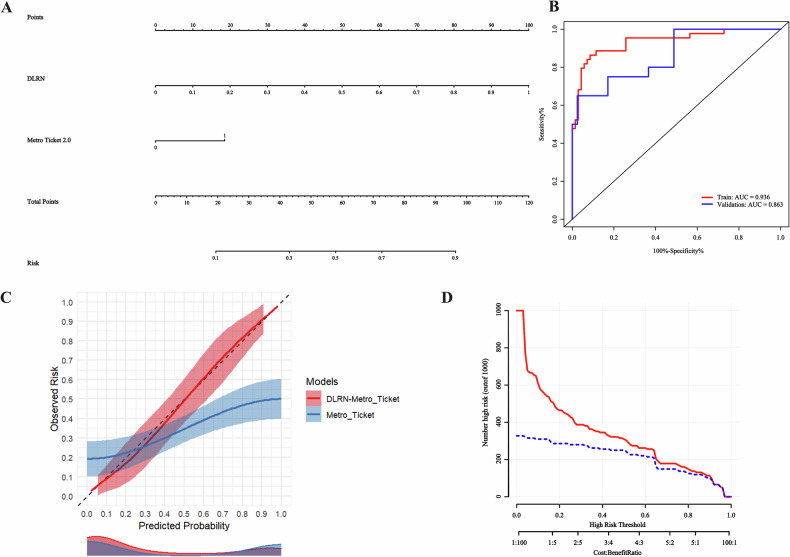
The nomogram (**A**), receiver operating characteristic curve (**B**), calibration curve (**C**), and clinical impact curve (**D**) of the DLRN-Metro-Ticket 2.0 were utilized to predict early recurrence of HCC post-LT. This DLRN-Metro-Ticket 2.0 nomogram integrates the DLRN score with the Metro-Ticket 2.0 model

The DLRN-Milan, DLRN-UCSF, DLRN-Metro-Ticket 2.0, and DLRN-Hangzhou nomograms, along with their respective ROC curves, are depicted in Figs. S6 and 5A, B. In the validation cohort, the AUC values for the DLRN-Milan, DLRN-UCSF, DLRN-Metro-Ticket 2.0, and DLRN-Hangzhou nomograms were 0.800, 0.790, 0.863, and 0.837, respectively. Notably, the DLRN-Metro-Ticket 2.0 nomogram demonstrated superior predictive performance, as evidenced by its calibration curve shown in Fig. 5C, with a Brier score of less than 0.250. Figure 5D illustrates the Clinical Impact Curve (CIC) of the DLRN-Metro-Ticket 2.0 model. The red curve represents the predicted number of individuals at high risk, while the blue curve indicates those predicted to be at high risk who subsequently experience early relapse. By comparing these curves, one can assess the model’s predictive accuracy and clinical applicability across various thresholds, thereby informing clinical decision-making. This DL study achieved a score of 41 points (67.21%) as detailed in the Supplementary Material RQS.

## Discussion

Despite stringent selection criteria, the incidence of HCC recurrence following LT remains high, predominantly occurring within the first two years post-transplantation. The prognosis following recurrence is poor, with a median survival of 12 months [22]. In this study, we constructed and validated the DLRN as a multimodal prediction tool for early recurrence after LT in HCC patients. Its clinical translational potential is reflected in its integration into the clinical decision-making framework of pre-transplant prognostic evaluation and post-operative dynamic monitoring, thereby optimizing individualized treatment strategies. Our study had two objectives: (a) to establish a DLRN based on preoperative multiphase CT in HCC patients undergoing LT and test the performance of the model in predicting early recurrence; (b) to further integrate the DLRN with traditional risk criteria to evaluate the incremental value and improve the prediction efficiency. The DLRN combining MVI, BCLC stage, AP peritumoral enhancement, and radiomics and DL scores had an AUC of 0.884 and 0.829 in the training/validation cohorts, respectively. The DLRN had a good effect on the RFS stratification of patients (*p* < 0.001). In addition, the nomogram integrating the DLRN with the Metro-Ticket 2.0 criteria performed best for all patients (AUC, training/validation: 0.936/0.863).

Unlike other tumors, HCC diagnosis primarily depends on imaging, confirmed by dynamic contrast-enhanced CT or MRI [23, 24]. For patients undergoing LT, preoperative imaging assesses liver volume and anatomical structures [25]. This study introduces a DL radiomics model using four-phase CT to predict early HCC recurrence post-LT. By leveraging multiphase CT’s ability to capture HCC’s hemodynamic changes, the model combines radiomics and DL features. Previous research has shown that multiphase CT radiomics effectively evaluates HCC chemotherapy response [26], pathological grading [9, 27], and MVI status [28]. Consistent with past findings [29–31], this study confirms the link between MVI, AP peritumoral enhancement, and HCC recurrence risk. To assess the added value of DL and radiomics features over clinical features in predicting early HCC recurrence post-LT, a clinical model incorporating MVI, AP peritumoral enhancement, and BCLC stage was enhanced with DL radiomics features to create a DLRN. The DLRN outperformed the clinical model, achieving a higher AUC (0.829 vs 0.670) and greater clinical net benefit in the validation cohort. Its AUC also surpassed previously reported clinical models (AUC: 0.733–0.785) [32]. Further validation in diverse patient groups is necessary to ensure the nomogram’s reliability, with recommendations to strengthen external validation through multi-institutional collaboration or larger databases.

Recent studies highlight the significant role of the peritumoral microenvironment in HCC progression and metastasis [33, 34]. Previous HCC radiomics studies have largely overlooked the peritumoral region, focusing instead on the tumor itself. However, recent findings indicate that radiomics features from the peritumoral area are closely linked to tumor progression processes like inflammation, neovascularization, fibrosis, and immune regulation [18]. Therefore, in this study, not only the radiomics and DL features of the tumor area were extracted for analysis, but also the peritumoral features were extracted as a supplement. Combining intratumoral and peritumoral data has been shown to improve predictions of HCC overall survival, achieving an AUC of 0.754–0.839 in tests [35, 36]. In line with these findings, the combination improved early recurrence prediction of HCC post-LT, highlighting the importance of tissue characteristics around the tumor for understanding invasion, metastasis, and recurrence potential.

Tumor heterogeneity (ITH) encompasses spatial and temporal differences within tumors [37]. Unlike traditional biopsy, imaging offers a non-invasive way to analyze disease evolution driven by pathophysiological mechanisms [38]. Wang et al demonstrated that MRI texture features combined with RNA sequencing are strongly linked to the infiltration of Apolipoprotein E^+^ macrophages and inflammatory cancer-associated fibroblasts [39]. In this study, 19 features were selected for constructing the RAD model, with 68.4% being high-order filters and wavelet texture features that, despite low interpretability, offer deep biological insights into ITH and are sensitive to early recurrence prediction. The RAD model achieved an AUC of 0.793 in the validation cohort, indicating good predictive value. DL captures deep ITH through neural network tasks and shows promise in predicting disease progression and prognosis in HCC patients [40, 41]. Radiomics features and DL features epitomize the analytical and data-driven paradigms of imaging data analysis, respectively. These paradigms are non-redundant and possess the potential for complementary integration. At the resolution scale of imaging modalities such as CT, they are instrumental in elucidating biological characteristics, such as ITH and tumor microenvironment reconstruction, which are pertinent to the prognosis of HCC. Nevertheless, due to the inherent limitations in spatial resolution, these features predominantly reflect heterogeneity at the macroscopic level of tissue and may not be capable of directly and precisely resolving microenvironmental changes at the cellular level. Our study found that DLRN outperformed RAD, DL, and clinical pathological models in predicting early recurrence of HCC after LT, achieving an AUC of 0.829 in the validation cohort. This confirms that integrating radiomics and DL features enhances individualized recurrence prediction accuracy.

Previous studies have also explored DL and radiomics for post-LT prognosis. Nie et al linked CT radiomics features to RFS in 196 HCC patients, achieving a *C* index of 0.749 [42]. Qu et al used DL on pathological sections from 380 HCC patients, achieving a *C* index of 0.794 [43]. Our study showed that DLRN, combined with clinical data, independently predicted early recurrence with a significantly improved HR estimate (*p* < 0.001), and high-risk patients had notably shorter RFS (log-rank *p* < 0.001). We stratified patients based on the Milan, UCSF, Metro-Ticket 2.0, and Hangzhou criteria (Fig. S4), improving stratification efficiency. We chose four criteria for our study: the Milan and UCSF criteria, due to their long-standing international recognition and comparability with global studies; and the Metro-Ticket 2.0 model and Hangzhou criteria, which include AFP thresholds for tumor specificity. We excluded China’s “Fudan Criteria” [44] because studies have shown no significant difference in recurrence rates compared to the Hangzhou criteria, which already considers biological characteristics [45]. HuaXi-eRPS outlines a surgical approach to prevent large size syndrome due to a mismatch between the graft and the recipient’s chest cavity size [46]. Our study excluded this criterion because the proposed predictor, involving graft weight and size ratios, is still in early research stages and requires further validation through large-scale trials. To assess the added value of DLRN, we created an integrated model combining DLRN with traditional systems, which significantly enhanced predictive efficiency. The DLRN and Metro-Ticket 2.0 model showed the best clinical consistency, with an AUC of 0.863 in the validation cohort. This study confirmed the compatibility of multimodal features with clinical parameters and supported improved LT candidate screening, postoperative follow-up, and treatment decisions, advancing precision management in HCC LT.

Our study has several limitations. Firstly, the main limitation is the risk of selection bias due to the retrospective study design and the limited sample. The small test cohort may hinder external validation and raise overfitting concerns. We optimized the model to enhance credibility, but its generalization across institutions, ethnic groups, and clinical scenarios requires further prospective multicenter studies. Secondly, MRI offers superior soft tissue contrast over CT, improving the analysis of HCC peritumoral microenvironment and enhancing imaging omics analysis through automated segmentation. Lastly, integrating tissue pathology images and combining imaging with genetic data could improve the DL model’s biological representation [47]. Multi-omics analysis reveals ITH, enhancing prognosis prediction.

CT-based DL radiomics offers non-invasive early recurrence prediction in HCC patients post-LT, outperforming clinical models and traditional scoring systems. It serves as a valuable tool for personalized HCC treatment with strong clinical potential.

## Supplementary information

ELECTRONIC SUPPLEMENTARY MATERIAL

## Data Availability

The datasets used and analyzed during the current study are available from the corresponding author upon reasonable request.

## Abbreviations

AFP: Alpha-fetoprotein
AI: Artificial intelligence
ALT: Alanine aminotransferase
AST: Aspartate aminotransferase
AUC: Area under the curve
BCLC: Barcelona Clinic Liver Cancer
CEA: Carcinoembryonic antigen
CK-19: Cytokeratin-19
DL: Deep learning
DLRN: Deep learning radiomics nomogram
HCC: Hepatocellular carcinoma
ITH: Tumor heterogeneity
LT: Liver transplantation
MELD: Model for end-stage liver disease
MVI: Microvascular invasion
RAD: Radiomic
ResNet: Residual convolutional network
RFS: Recurrence-free survival
SVM: Support vector machine
UCSF: University of California, San Francisco
VOI: Volume of interest
