# Supplementary material for: Augmenting conventional criteria: a CT-based deep learning radiomics nomogram for early recurrence risk stratification in hepatocellular carcinoma after liver transplantation
Source: Insights Imaging. 2025 Sep 17;16:194. doi: 10.1186/s13244-025-02082-7 (PMC12443646; doi:10.1186/s13244-025-02082-7)
Supplement: Supplementary file 1 — ELECTRONIC SUPPLEMENTARY MATERIAL [file 13244_2025_2082_MOESM1_ESM.pdf]

Augmenting Conventional Criteria: A CT-Based Deep Learning Radiomics Nomogram for Early Recurrence Risk Stratification in Hepatocellular Carcinoma After Liver Transplantation

ELECTRONIC SUPPLEMENTARY MATERIAL

This supplementary material includes:

1. Supplementary Methods

E1.1 The inclusion and exclusion criteria

E1.2 Classic liver transplantation surgical approach

E1.3 CT study protocols

E1.4 Definition of radiologic features

E1.5 Image registration

E1.6 Image resampling and discretization

E1.7 Radiomic and deep learning feature extraction

E1.8Feature selection and model building

E1.9 RAD-score and DL-score formula

2. Supplementary Figures

Figure S1 Patient recruitment pathway

Figure S2 The representative axial CT images of radiologic features

Figure S3 Architecture of ResNet-18

Figure S4 Decision curve and performance comparison of different models for HCC early recurrence prediction.

Fig. S5 Kaplan-Meier curves of recurrence-free survival (RFS) by deep learning radiomics nomogram (DLRN) score in subgroups

Fig. S6 Deep learning radiomics nomogram (DLRN)-traditional risk standards nomograms for predicting early recurrence of HCC after LT and their receiver operating characteristic curves

3. Supplementary Tables

Table S1. CT scan protocols

Table S2. CT Image filtering preprocessing methods and explanation

Table S3. Univariate analysis of the groups with and without early recurrence in the training set

Table S4. Multivariate regression analysis of the groups with and without early recurrence in the training set

Table S5. Selected features included in five radiomics models

Table S6. Area under the receiver operating characteristic curve of a radiomics model for predicting early recurrence of hepatocellular carcinoma after liver transplantation

Table S7. Collinearity analysis of clinical factors with RAD and DL signature

SUPPLEMENTARY MATERIAL RQS

1. Supplementary Methods

E1.1 The inclusion and exclusion criteria

The inclusion criteria were listed as follows: i) age 18-70 years old; ii) HCC confirmed by pathology after LT; iii) receiving enhanced CT scan within 15 days before LT; iv) CT image quality was good and could be used for analysis; v) complete clinicopathological and follow-up data were available; vi) classic liver transplantation.

The exclusion criteria were as follows: i) presence of other pathological types, such as intrahepatic cholangiocarcinoma (ICC) or mixed hepatocellular cholangiocarcinoma (CHC); ii) death or disease recurrence within 1 month after liver transplantation.

E1.2 Classic liver transplantation surgical approach

For diseased liver resection, cut the perihepatic ligament, then sequentially dissect the first and second hepatic hilum and the inferior vena cava. The first hepatic hilum dissection should be close to the liver, with the bile duct cut above the cystic duct level. Dissect the hepatic artery along the proper hepatic artery, freeing and cutting the left and right hepatic arteries individually. To match the recipient's hepatic artery with the donor's, the common hepatic artery is typically separated 1 to 2 cm downward, and the gastroduodenal artery is cut. The portal vein is exposed by gently separating the artery and bile duct, then isolated from surrounding tissue up to the liver hilum and down to the pancreas. In cases of portal vein thrombosis, further separation to the splenic vein confluence is done for thrombus removal. The three hepatic veins at the second liver portal remain intact, while the superior and inferior vena cava are fully freed. Phrenic veins are carefully separated, ligated, and cut, and the surrounding diaphragmatic tissue is cleared to expose the vena cava for anastomosis. Free the retrohepatic inferior vena cava's posterior wall. Separate the infrahepatic inferior vena cava above the renal vein. Once the donor liver is trimmed, block and cut the portal vein near the liver portal. Simultaneously, block and cut the suprahepatic and infrahepatic vena cava near the liver, removing the diseased liver and

attached vena cava. Check for bleeding and prepare vessels for anastomosis. For donor liver implantation, connect the donor's and recipient's superior and inferior vena cava end-to-end, ensuring smooth sutures. Before closing the anterior wall, irrigate the donor liver with 800 mL of 4℃ isotonic NaCl solution with 5% albumin to clean residual preservation fluid. Air is removed from the inferior vena cava. The donor liver's portal vein is connected end-to-end with the recipient's portal vein, ensuring proper length to avoid twisting or tension. Avoid tying a knot at the suture's end. Use a blocking clamp on the donor portal vein above the anastomosis, open the recipient's portal vein, and release 200-300 mL of blood. Check blood potassium levels before restoring blood flow; treat high levels promptly to prevent cardiac arrest. Sequentially open the superior and inferior vena cava, and the portal vein, then flush the liver with warm isotonic NaCl to rewarm it. If blood stasis occurs, gently massage the liver to aid circulation. If the donor liver functions well, bile should flow within minutes. Begin arterial reconstruction after ensuring no active bleeding. The donor's common hepatic artery is typically trimmed for end-to-end anastomosis with the recipient's common hepatic and gastroduodenal arteries. After vascular anastomosis, intraoperative ultrasound ensures the hepatic, portal, and hepatic veins are unobstructed. If bile ducts are in good condition, end-to-end anastomosis is used for reconstruction. The donor bile duct is trimmed to a well-supplied area above the cystic duct confluence, while the recipient's duct is trimmed only at ischemic edges. The anastomosis should be tension-free. Before anastomosis, remove the gauze, loosen the retractor, and position the liver naturally. Ensure hemostasis, place drainage tubes, and close the abdomen in layers.

E1.3CT study protocols

After obtaining plain images, iodinated contrast agents, including iodixanol (Ultravist 300 or Ultravist 370, Bayer, Germany) or ioversefol (Optiray 320 or Optiray 350, Guerbet, France) were administered intravenously at a flow rate of 3.0–4.0 mL/s at a volume of 1.3–1.5 mL/kg body weight. Arterial, portal venous, and delayed phase images were obtained at 25–30 seconds, 60 seconds, and 150–180 seconds after contrast injection.

E1.4 Definition of radiologic features

i. Number of tumors: Tumor counts were performed during the arterial or portal venous phase.

ii. Maximum tumor diameter: The maximum long diameter of the tumor measured on the transverse axial image during the portal venous or delayed phase.

iii. Tumor margin: Clear margins are characterized by a clear boundary between the tumor and the surrounding liver parenchyma, with sharp borders and regular morphology; unclear margins are characterized by a blurred boundary between the tumor and the surrounding liver parenchyma, with a transitional area. Tumors are irregular in morphology, with jagged, burr-like edges or locally convex nodules.

iv. Peritumor enhancement during the arterial phase: A high-density area appears outside the tumor outline during the arterial phase.

v. Pseudocapsule: A high-density ring appears at the edge of the tumor during the portal venous or delayed phase.

Fig.S2 shows representative CT images of radiological features

E1.5 Image registration

In this study, Elastix software (https://github.com/SuperElastix/ElastixModelZoo) was used to perform non-affine transformation on non-contrast phase (N), arterial phase (A), and delayed phase (D) images using portal venous phase (V) images as reference images to ensure that these images were consistent with portal venous phase images in terms of image information parameters.

E1.6 Image resampling and discretization

Images were resampled to a voxel size of 1 × 1 × 1 mm to standardize voxel spacing and the number of bins was set to 25 to discretize the total distribution.

E1.7 Radiomic and deep learning feature extraction

Radiomic features were not extracted only from raw images. Features were also obtained from nine types of filtered images, including exponential filtering, gradient filtering, two-dimensional local binary pattern (LBP-2D), three-dimensional local binary pattern (LBP-3D), logarithmic filtering, square filtering, square root filtering, and wavelet filtering. According to the standards of the Imaging Biomarker Standardization Initiative, a total of 4960 radiomic features were extracted, covering first-order features, shape-based features, and texture features (such as gray-level co-occurrence matrix, gray-level run-length matrix, gray-level size area matrix, adjacent gray-level difference matrix, and gray-level dependence matrix features). Considering the physical significance of the artificially generated annular peritumoral volume of interest, we only extracted raw image features. In addition, the feature values were normalized to a normal distribution by the Z-score method.

The structure of ResNet-18 is shown in **Fig. S3**. It starts with a 7x7 convolutional layer, followed by 4 residual modules. Each residual module has 4 convolutional layers. These 17 convolutional layers plus the final fully connected layer make a total of 18 layers, so it is called ResNet-18. Each part of ResNet-18 has a specific function: i) Input module: receiving and preprocessing image data; ii) First layer of convolution and pooling: preliminarily extracting low-level features and downsampling the image; iii) Residual block group: extracting higher-level features layer by layer, and solving the gradient vanishing problem of deep networks through residual learning; iv) Global average pooling layer: compressing feature maps into fixed-size feature vectors; v) Fully connected layer: mapping feature vectors to category labels to complete classification tasks[1]. The feature extraction capability of ResNet-18 is achieved through network self-learning rather than manual design, and is independent of specific tasks. It has been widely used in computer vision tasks such as image classification, object localization, and object detection. In this study, deep learning feature extraction was achieved through the following steps: first, the CT image was adjusted to the soft tissue window width (W=350) and window level (L=50), and then the image grayscale value was mapped to the range [0, 255]. The preprocessed image was input into the ResNet18 model pre-trained based on the PyTorch framework to achieve feature extraction. A total of 4096 features were extracted from the tumor (VOI_{Tumor}) and the peritumoral volume of interest ($VOI_{Peritumor}$) at each stage.

E1.8 Feature selection and model building

The selection of radiomics and deep learning features was mainly divided into three steps: i) The Mann-Whitney U test was used to compare the differences in radiomics features between the early recurrence group and the non-early recurrence group, and the features with $P < 0.05$ were screened as significant features; ii) The correlation between features was evaluated by Spearman correlation analysis, and for feature pairs with correlation coefficients higher than 0.90, the features with lower importance weights

were removed; iii) The least absolute shrinkage and selection operator (LASSO) algorithm was used to select features with non-zero coefficients to reduce the impact of multicollinearity.

E1.9 RAD-score and DL-score formula

RAD-score = -1.75234564644120 + (0.346371678587235) * original_glszm_SmallAreaEmphasis.NP peritumor + (0.618500661255596) * original_glszm_SmallAreaHighGrayLevelEmphasis.AP peritumor +(0.0598086062608929) * original_glcm_Idn.VP peritumor + (0.386253240510915) * original_glszm_SmallAreaHighGrayLevelEmphasis.VP peritumor + (-0.315064757059355) * original_ngtdm_Contrast.AP tumor + (-1.25803918913479) * log-sigma-3-mm-3D_firstorder_RootMeanSquared.AP tumor + (-1.56001868812426) * wavelet-HLL_glcm_ClusterShade.AP tumor + (0.304585017615633) * wavelet-HLL_glcm_MCC.AP tumor + (-0.312627695360976) * wavelet-HLL_glszm_GrayLevelNonUniformityNormalized.AP tumor + (-0.031722117660896) * wavelet-HHL_glszm_SmallAreaLowGrayLevelEmphasis.AP tumor + (-0.197582717443648) * log-sigma-3-mm-3D_ngtdm_Contrast.DP tumor + (-0.566827210047355) * wavelet-HLL_firstorder_Skewness.DP tumor + (0.0590066018377759) * wavelet-HLL_glcm_MCC.DP tumor + (-0.106616162662847) * wavelet-HLL_glszm_GrayLevelNonUniformityNormalized.DP tumor + (-0.105561608331654) * wavelet-HHL_glszm_SmallAreaLowGrayLevelEmphasis.DP tumor + (0.044013499934984) * original_glszm_SmallAreaHighGrayLevelEmphasis.NP tumor + (0.245391592155988) * log-sigma-1-mm-3D_glszm_SmallAreaEmphasis.NP tumor + (0.0610606544318534) * log-sigma-1-mm-3D_glszm_GrayLevelNonUniformityNormalized.VP tumor + (0.646922842075291) * wavelet-HHH_firstorder_Kurtosis.VP tumor

DL-score =-0.241208216350357+(0.32547062335402)* feature_1.AP peritumor+(0.447471387962046)* feature_86.AP peritumor +(0.70473784909323)* feature_120.AP peritumor+(0.0963348304230041)* feature_280.AP peritumor+(0.0332082187622064)* feature_460.AP peritumor (0.0207278691433518)* feature_141.DP peritumor+(0.120706340616862)* feature_466.DP peritumor+(0.246643638193884)* feature_1.NP peritumor+(0.0719149559288065)* feature_215.AP tumor+(0.244300899647419)* feature_169.DP tumor +(0.0170101365332128)* feature_180.DP tumor

2. Supplementary Figures

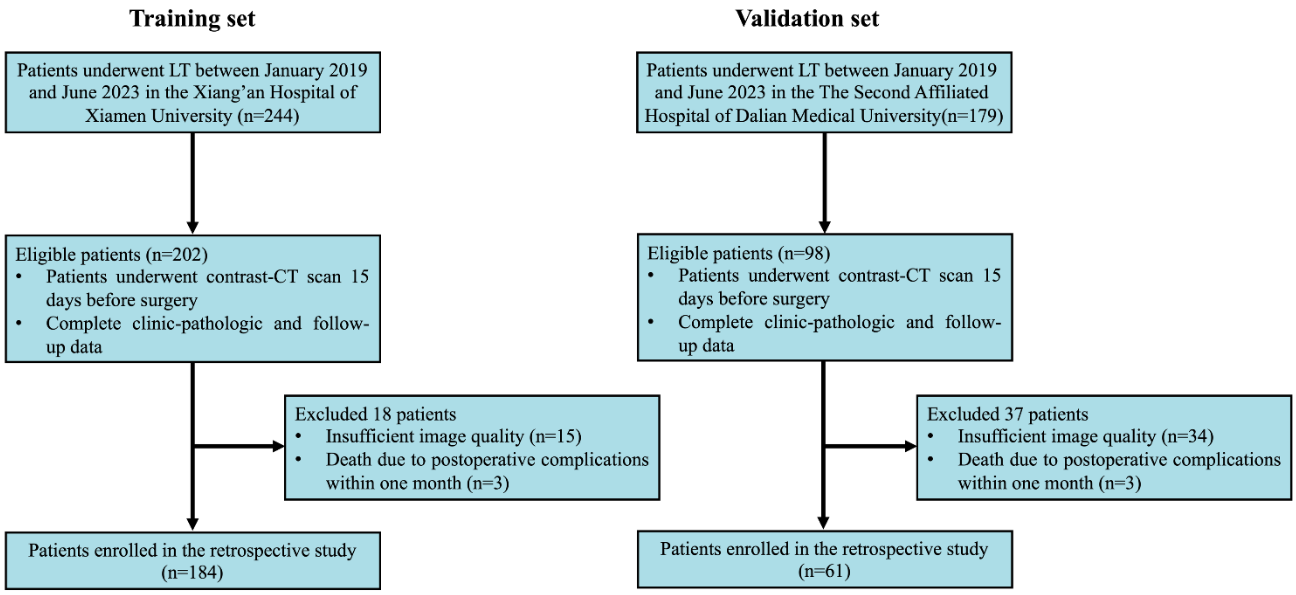

Fig.S1 Patient recruitment pathway.

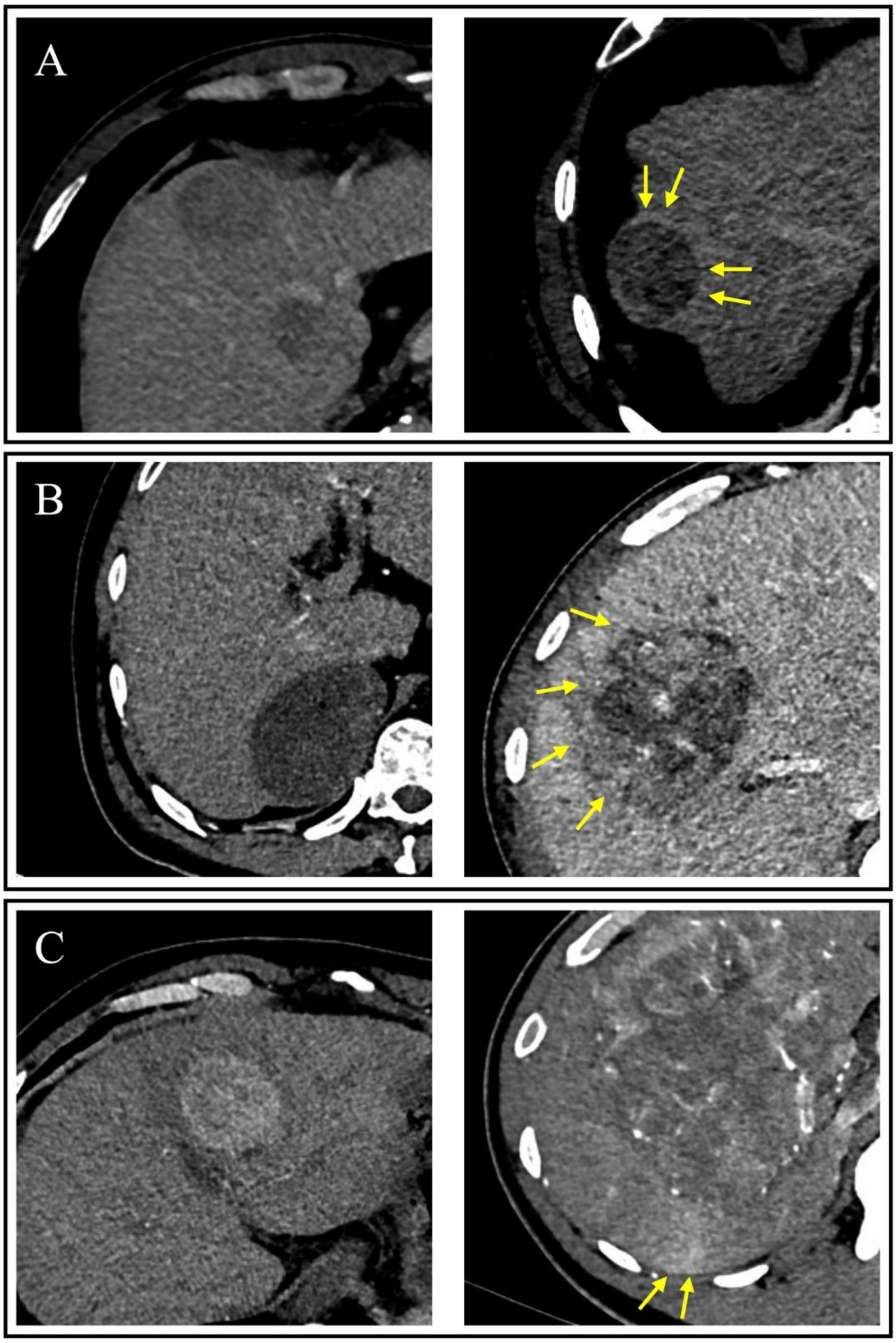

Fig S2 Representative axial CT images of radiological features. The right columns of (**A–C**) show a well-defined pseudocapsule, rough margins, and arterial phase peritumoral enhancement (marked by yellow arrows), respectively. The left columns of (**A–C**) show an ill-defined pseudocapsule, smooth margins, and no arterial phase peritumoral enhancement, respectively.

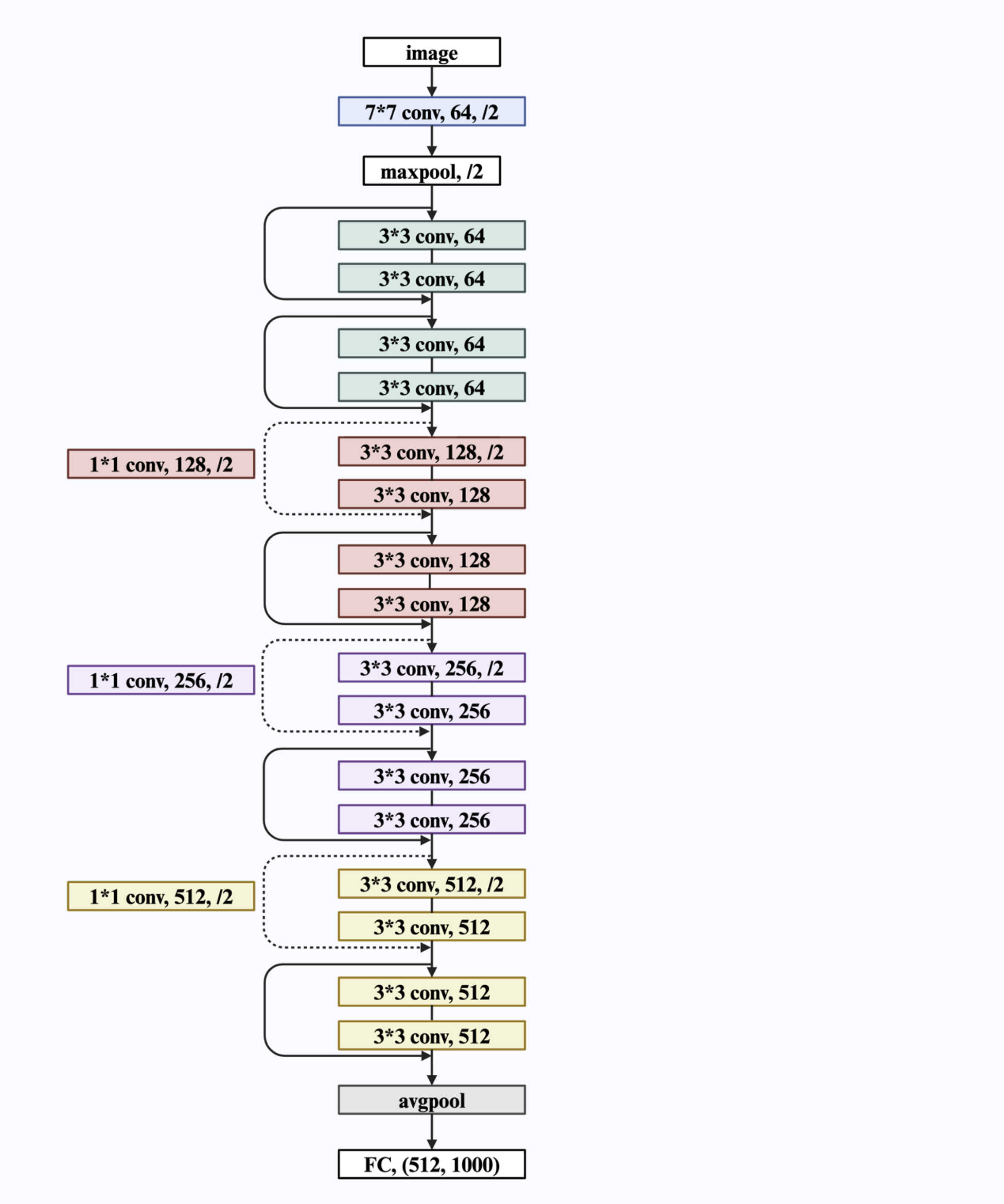

Fig. S3 Architecture of ResNet-18

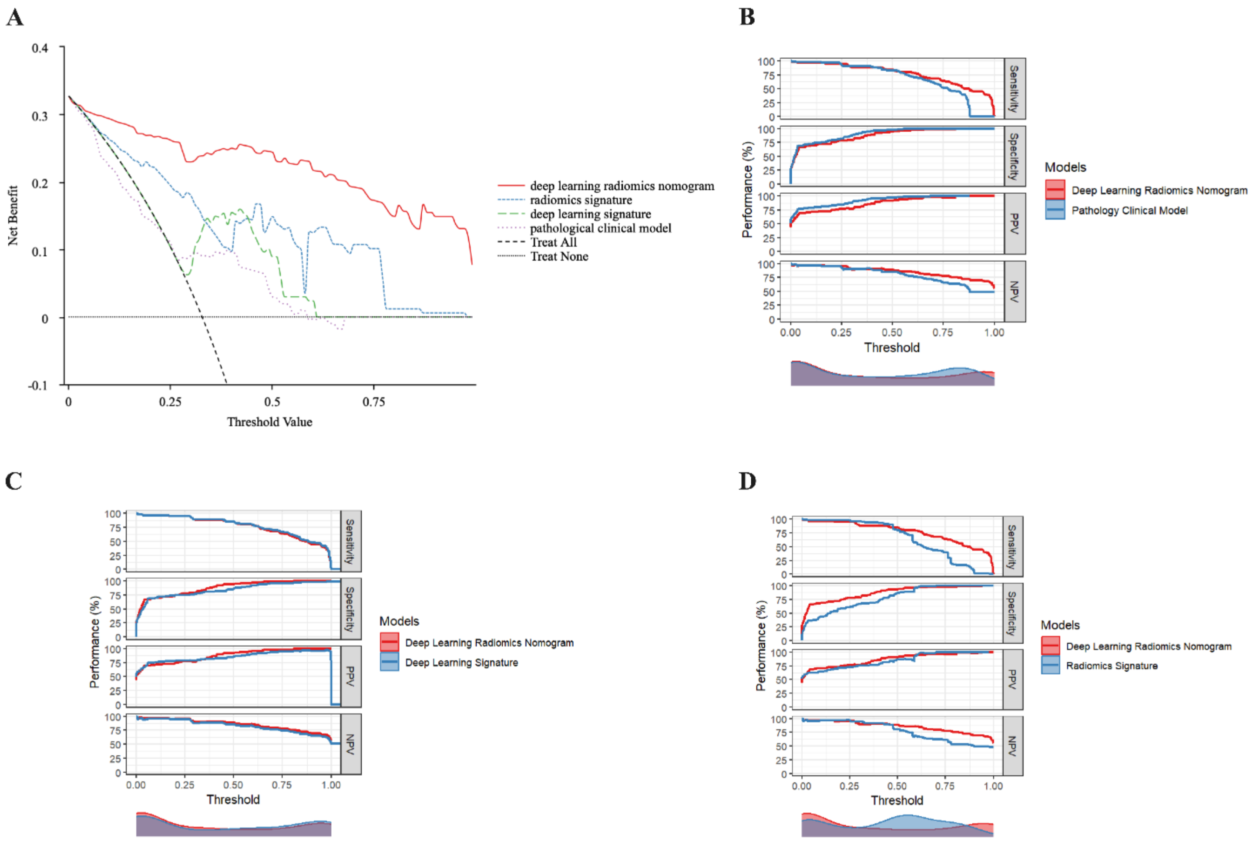

Fig. S4 Decision curve and performance comparison of different models for HCC early recurrence prediction. PPV, positive predictive value；NPV, negative predictive value.

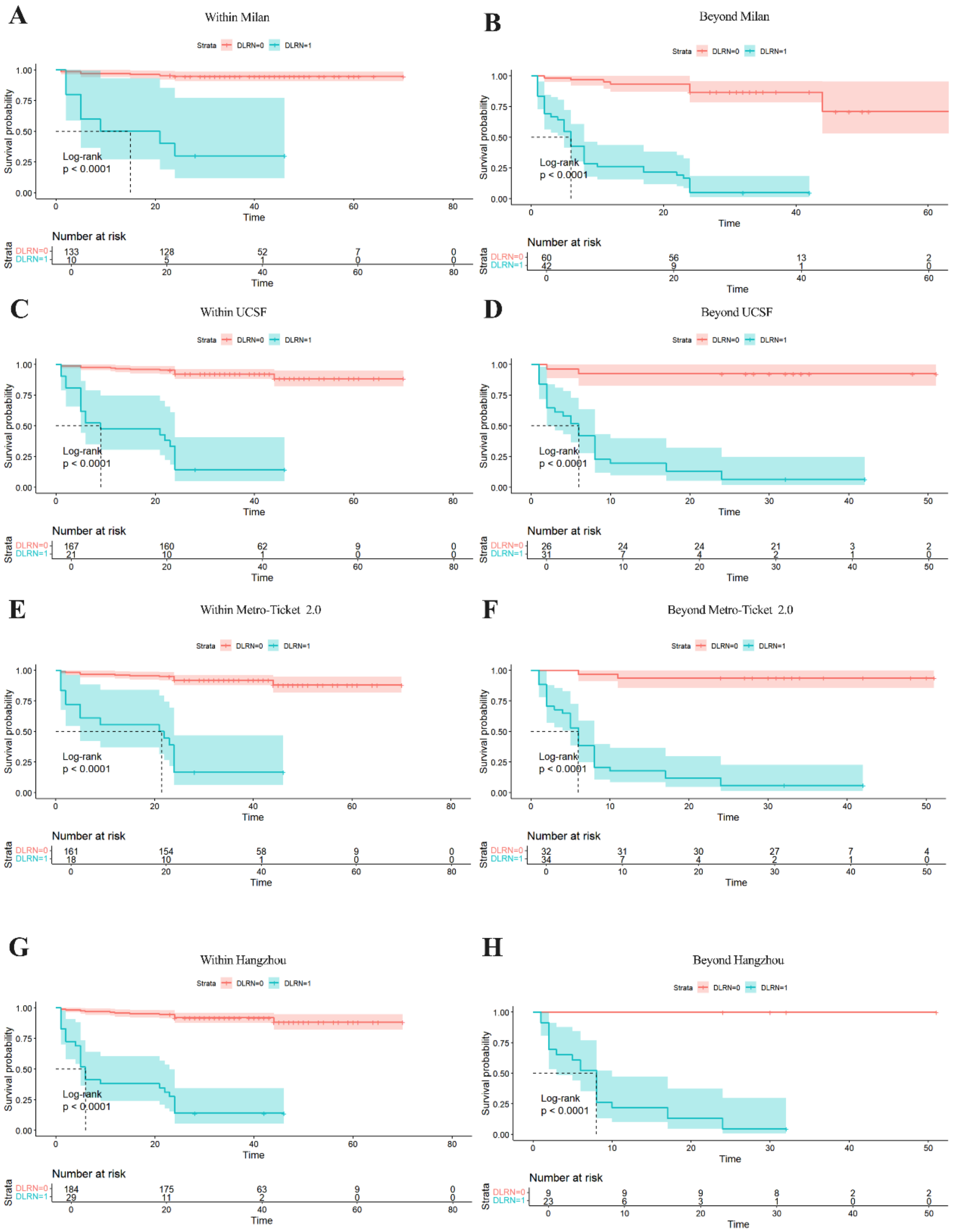

Fig. S5 Kaplan-Meier curves of recurrence-free survival (RFS) by deep learning radiomics nomogram (DLRN) score in subgroups stratified by Milan, University of California, San Francisco (UCSF), Metro-Ticket 2.0, and Hangzhou criteria. (**A**) Within Milan criteria; (**B**) Beyond Milan criteria; (**C**) Within UCSF criteria; (**D**) Beyond UCSF criteria; (**E**) Within Metro-Ticket 2.0 criteria; (**F**) Beyond Metro-Ticket 2.0 criteria; (**G**) Within Hangzhou criteria; (**H**) Beyond Hangzhou criteria.

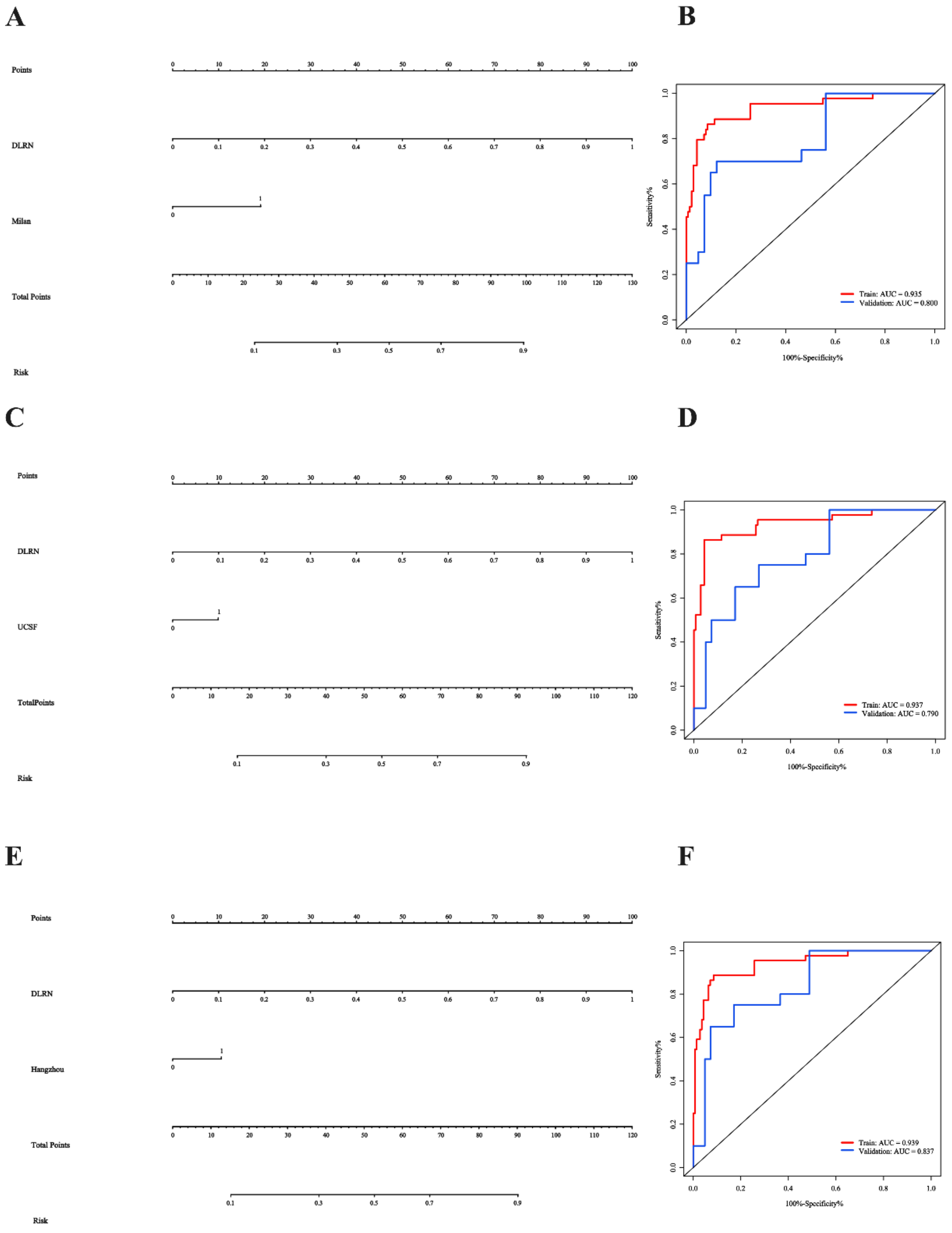

Fig. S6 Deep learning radiomics nomogram (DLRN)-Milan (**A**), DLRN-UCSF (University of California, San Francisco, **C**), and DLRN-Hangzhou (**E**) nomograms for predicting early recurrence of HCC after LT and their receiver operating characteristic curves (**B, D, F**). The DLRN-Milan, DLRN-UCSF, and DLRN-Hangzhou nomograms combined the DLRN score with the Milan, UCSF, and Hangzhou criteria, respectively.

3. Supplementary Tables

Table S1. CT scan protocols

**CT scanner**	**CT 128**	**CT 256**	**CT 256**
**Scanner model**	Somatom Force	Revolution	Brilliance iCT 256
**Manufacturer**	SIEMENS	General Electric	Philips
**Gantry rotation time (s)**	0.25	0.28	0.5
**Tube voltage (kV)**	120	120	120
**Tube current**	Ref. 200 mAs (Care Dose 4D)	250-400 mA (automatic tube current modulation)	220mA
**Detector collimation (mm)**	0.6	0.625	0.625
**Matrix**	512×512	512×512	512×512
**Pitch**	1.0	0.992	1.0
**Slice thickness (mm)**	1	1	1
**Corresponding hospital**	a, b	b	b

Note: s (second); kV (kilovolt); mA (milliampere); mm (millimeter); a. Xiang'an Hospital of Xiamen University; b. The Second Affiliated Hospital of Dalian Medical University

Table S2. CT Image filtering preprocessing methods and explanation

Image filter (10 types)	Explanation
Original	No filter applied
Wavelet	Wavelet filtering results in 8 decompositions per level, considering all possible combinations of applying either a High or Low pass filter across the three dimensions.
LoG	The Laplacian of Gaussian filter is an edge enhancement filter that highlights regions with changes in gray levels, with sigma determining the coarseness of the emphasized texture. A low sigma value highlights fine textures that change over short distances, while a high sigma value highlights coarse textures with gray level changes over larger distances.
Square	The method involves squaring the image intensities and then linearly scaling them back to their original range, ensuring that any negative values in the original image remain negative after applying the filter.
SquareRoot	Calculates the square root of the absolute values of image intensities and rescales them to their initial range. Any negative values in the original image will be restored to negative after the filter is applied.
Logarithm	The logarithm of the absolute intensity plus one is taken. After applying the filter, values are rescaled to their original range, and any originally negative values are reverted to negative.
Exponential	Applies the exponential function, with the filtered intensity being e raised to the power of the absolute intensity. The values are then rescaled to their original range, and any originally negative values are reverted to negative after filtering.
Gradient	Outputs the gradient's magnitude.
LBP2D	Computes and provides a local binary pattern used in two dimensions.
LBP3D	Generates and provides local binary pattern maps in 3D using spherical harmonics, with the final image being the kurtosis map.

Table S3. Univariate analysis of the groups with and without early recurrence in the training set

Variables	Early recurrence (n = 44)	Without early recurrence (n = 140)	Difference analysis P-value	Univariate analysis OR (95%CI)	P-value
**Age (years)**	50.00 (42.50-55.50)	53.00 (47.75-56.00)	0.096	1.038 (1.01-1.08)	0.040*
**Gender**			0.230		0.237
Male	40 (90.9)	117 (83.6)			
Female	4 (9.1)	23 (16.4)			
**Etiology**			0.497		0.587
HBV	40 (90.9)	121 (86.4)			
HCV	1 (2.3)	2 (1.4)			
Others	3 (6.8)	17 (12.1)			
**Liver cirrhosis**			0.046		0.369
Absent	12 (27.3)	17 (12.1)			
Present	32 (72.7)	123 (87.9)			
**BCLC stage**			<0.001		
0+A	13 (29.6)	92 (65.7)			
B	18 (40.9)	31 (22.1)			
C	12 (27.3)	6 (4.3)			
D	1 (2.3)	11 (7.9)		21.80 (2.24-203.59)	0.008*
**AFP (ng/ml)**	31.99 (3.42-1210.00)	7.43 (2.95-88.30)	0.036	1.00 (1.00-1.01)	0.040
**ALT (U/L)**	31.00 (24.00-56.00)	26.15 (17.40-42.20)	0.046		0.562
**AST (U/L)**	51.00 (34.55-77.70)	39.00 (27.90-60.03)	0.024		0.735
**CEA (ng/ml)**	2.35 (1.67-3.30)	2.24 (1.23-3.87)	0.913		0.290
**MELD score**	10.00 (7.00-14.25)	9.00 (5.75-14.00)	0.403		0.386
**Child-Pugh**			0.954		0.954
A	23 (52.3)	75 (53.6)			
B	19 (43.2)	60 (42.9)			
C	2 (4.5)	5 (3.6)			
**Ki-67 PI**			0.009	4.56 (1.33-15.63)	0.016*

Ki-67≤ 10%	3 (6.8)	35 (25.0)			
Ki-67> 10%	41 (93.2)	105 (75.0)			
**CK-19**			0.746		0.746
Absent	31 (70.5)	95 (67.9)			
Present	13 (29.5)	45 (32.1)			
**MVI**			<0.001	3.60 (1.78-7.29)	<0.001*
Absent	20 (45.5)	105 (75.0)			
Present	24 (54.5)	35 (25.0)			
**Pre-LT treatment**			0.940		0.939
Absent	12 (27.3)	39 (27.9)			
Present	32 (72.7)	101 (72.1)			
**Histologic grade**			0.012		
Well differentiated	17 (38.6)	26 (18.6)			
Moderately differentiated	22 (50.0)	79 (56.4)			
Poorly differentiated	5 (11.4)	35 (25.0)		3.29 (1.13-9.61)	0.042*
**Tumor number**			0.108		0.111
Single	12 (27.3)	57 (40.7)			
Multiple	32 (72.7)	83 (59.3)			
**Maximum tumor diameter (cm)**	6.50 (4.38-11.00)	2.80 (1.58-5.80)	<0.001	1.86 (1.80-1.92)	<0.001*
**Tumor borderline, clear**			0.039		0.051
Absent	27 (61.4)	61 (43.6)			
Present	17 (38.6)	79 (56.4)			
**AP peritumoral enhancement**			0.009	2.55 (1.24-5.21)	0.011*
Absent	14 (31.8)	76 (54.3)			
Present	30 (68.2)	64 (45.7)			
**Pseudocapsule, well-defined**			0.498		0.375
Absent	7 (15.9)	31 (22.1)			
present	37 (84.1)	109 (77.9)			

Note: Data are presented as number (%) or median (interquartile range, IQR). OR, odds ratio; CI, confidence interval; HBV, hepatitis B virus; HCV, hepatitis C virus; AFP, alpha-fetoprotein; ALT, alanine amino-transferase; AST, aspartate amino-transferase; CEA, carcinoembryonic antigen; BCLC, barcelona clinic liver cancer; PI, proliferation index; CK, cytokine; AP, arterial phase; MVI, microvascular invasion; LT, liver transplantation; Pre-LT treatment strategies included surgery, ablation, transarterial chemoembolization, chemotherapy, and targeting therapy. * P<0.05.

Table S4. Multivariate regression analysis of the groups with and without early recurrence in the training set

Variables	OR (95%CI)	P-value
**Age**	1.03 (0.98-1.07)	0.229
**BCLC stage**	9.30 (3.00-58.10)	0.019*
**Ki-67 PI**	4.34 (1.08-14.48)	0.059
**MVI**	3.96 (1.53-10.24)	0.005*
**Histologic grade**	1.79 (1.25-2.48)	0.689
**Maximum tumor diameter**	1.04 (0.93-1.17)	0.520
**AP peritumoral enhancement**	2.86 (1.14-7.21)	0.026*

Note: OR, odds ratio; CI, confidence interval; BCLC, barcelona clinic liver cancer; PI, proliferation index; AP, arterial phase; MVI, microvascular invasion; * P<0.05.

Table S5. Selected features included in five radiomics models

Radiomics models	Selected features
**Tumor (n = 13)**	log-sigma-1-mm-3D_glrlm_HighGrayLevelRunEmphasis.NP tumor<br>original_shape_MajorAxisLength.AP tumor<br>original_firstorder_Maximum.AP tumor<br>log-sigma-1-mm-3D_firstorder_MeanAbsoluteDeviation.AP tumor<br>wavelet-HLL_glcm_ClusterShade.AP tumor<br>wavelet-HLL_gldm_SmallDependenceHighGrayLevelEmphasis.AP tumor<br>wavelet-HHL_glszm_ZoneEntropy.AP tumor<br>original_ngtdm_Strength.VP tumor<br>log-sigma-1-mm-3D_ngtdm_Strength.VP tumor<br>wavelet-LLH_glcm_JointEntropy.VP tumor<br>original_glcm_Contrast.DP tumor<br>log-sigma-2-mm-3D_glszm_GrayLevelNonUniformity.DP tumor<br>wavelet-HLL_glszm_GrayLevelVariance.DP tumor
**Peritumor (n = 6)**	original_glszm_SmallAreaEmphasis.NP peritumor<br>original_shape_LeastAxisLenfth.AP peritumor<br>original_firstorder_Minimum.AP peritumor<br>original_gldm_SmallDependenceHighGrayLevelEmphasis.VP peritumor<br>original_ngtdm_Complexity.VP peritumor<br>original_firstorder_Maximum.DP peritumor
**Tumor+Peritumor (n = 19)**	original_glszm_SmallAreaEmphasis.NP peritumor<br>original_glszm_SmallAreaHighGrayLevelEmphasis.NP tumor<br>log-sigma-1-mm-3D_glszm_SmallAreaEmphasis.NP tumor

	original_glszm_SmallAreaHighGrayLevelEmphasis.AP peritumor<br>original_ngtdm_Contrast.AP tumor<br>log-sigma-3-mm-3D_firstorder_RootMeanSquared.AP tumor<br>wavelet-HLL_glcm_ClusterShade.AP tumor<br>wavelet-HLL_glcm_MCC.AP tumor<br>wavelet-HLL_glszm_GrayLevelNonUniformityNormalized.AP tumor<br>wavelet-HHL_glszm_SmallAreaLowGrayLevelEmphasis.AP tumor<br>original_glcm_Idn.VP peritumor<br>original_glszm_SmallAreaHighGrayLevelEmphasis.VP peritumor<br>log-sigma-1-mm-3D_glszm_GrayLevelNonUniformityNormalized.VP tumor<br>wavelet-HHH_firstorder_Kurtosis.VP tumor<br>log-sigma-3-mm-3D_ngtdm_Contrast.DP tumor<br>wavelet-HLL_firstorder_Skewness.DP tumor<br>wavelet-HLL_glcm_MCC.DP tumor<br>wavelet-HLL_glszm_GrayLevelNonUniformityNormalized.DP tumor<br>wavelet-HHL_glszm_SmallAreaLowGrayLevelEmphasis.DP tumor

Note： N = noncontrast, A = arterial, V = portal venous, D = delayed.

Table S6. Area under the receiver operating characteristic curve of a radiomics model for predicting early recurrence of hepatocellular carcinoma after liver transplantation

Model	Training	Validation
**Peritumoral radiomics models**		
SVM	0.808（0.734, 0.882）	0.742 （0.617, 0.866）
LightGBM	0.828（0.761, 0.895）	0.728（0.588, 0.868）
XGBoost	0.756（0.655, 0.858）	0.737（0.605, 0.868）
Random Forest	0.868（0.799, 0.937）	0.664（0.501, 0.827）
SGD	0.774（0.695, 0.853）	0.681（0.544, 0.817）
**Tumor radiomics models**		
SVM	0.829（0.757, 0.900）	0.744（0.613, 0.875）
LightGBM	0.769（0.683, 0.855）	0.722（0.594, 0.850）
XGBoost	0.787（0.693, 0.882）	0.633（0.465, 0.801）
Random Forest	0.866（0.815, 0.918）	0.690（0.539, 0.841）
SGD	0.787（0.713, 0.861）	0.667（0.498, 0.836）
**Fusion radiomics models**		
SVM	0.838（0.778, 0.897）	0.767（0.626, 0.908）
LightGBM	0.753（0.681, 0.826）	0.613（0.467, 0.760）
XGBoost	0.730（0.658, 0.802）	0.709（0.574, 0.844）
Random Forest	0.798（0.730, 0.865）	0.717（0.565, 0.868）
SGD	0.806（0.731, 0.880）	0.695（0.557, 0.833）

Note: Data in parentheses are 95% CIs. SVM, Support Vector Machine; LightGBM, Light Gradient Boosting Machine; XGBoost, Extreme Gradient Boosting; SGD, Stochastic Gradient Descent

Table S7. Collinearity analysis of clinical factors with RAD and DL signature

**Variables**	Collinearity Statistics	
	Tolerance	VIF
**Age**	0.905	1.106
**BCLC stage**	0.710	1.408
**Ki-67 PI**	0.884	1.131
**MVI**	0.789	1.267
**Histologic grade**	0.843	1.187
**Maximum tumor diameter**	0.623	1.604
**AP peritumoral enhancement**	0.827	1.209
**RAD signature**	0.870	1.149
**DL signature**	0.853	1.173

Note: VIF, variance inflation factors; BCLC, barcelona clinic liver cancer; PI, proliferation index; AP, arterial phase; MVI, microvascular invasion; RAD, radiomic; DL, deep learning.

[1] HE K Z, XIANGYU; REN, SHAOQING; SUN, JIAN. Deep Residual Learning for Image Recognition [Z]. IEEE Conference on Computer Vision and Pattern Recognition. Las Vegas, NV, USA; IEEE. 2016: 770-8.10.1109/CVPR.2016.90

SUPPLEMENTARY MATERIAL RQS

**Unmet clinical need (UCN) defined**<br><br>**O Uni-centre o Multi-centre**<br>**O International Multi-centre**
**Classification of the model: diagnostic, theragnostic, predictive, prognostic, follow-up**<br><br>**o Defined**<br>**O Not Clearly Defined**
**Input from Clinicians for interpretable pipeline development. Discussion regarding choosing appropriate explainability method**<br><br>◉ **Clinical knowledge incorporated in the pipeline or explainability method decided and agreed with the clinician before model development**<br>○ **Clinical knowledge not incorporated in the pipeline or explainability method not decided or agreed with the clinician before model development**

**Image protocol quality to be documented following the TRIAC level (Transparent Reporting of Medical Image Acquisition for a future proof radiomics). TRIAC guidelines describe five different levels of evidence for reporting imaging protocols. Level 0 indicates that the protocol has not been formally approved with a reference number; Level 1 indicates that the protocol has been approved with a reference number in the archive of the department; Level 2 indicates that the protocol has been approved with formal quality assurance (recommended minimum level for prospective trials); Level 3 indicates that the protocol is established internationally and has been published in guideline documents and peer-reviewed papers; Level 4 indicates that the protocol is Future proof i.e., the protocol follows TRIAC Level 3, FAIR principles and retains raw data.**<br><br>☑ **Protocols are well documented** ☑ **Public protocol is used**
**Hardware's used described, image reconstruction method specified**

☑ Description of the hardware used for image acquisition<br>☑ Information about image reconstruction method e.g. convolutional kernel
Preprocessing of the images<br><br>o Accounted for variation in slice thickness/ convolution kernel/ contrast<br>O Not accounted for variation in slice thickness/ convolution kernel/ contrast
Imaging at multiple time points - collect individuals' images at additional time points. Analyze feature robustness to temporal variabilities (e.g., organ movement, organ expansion/shrinkage)<br><br>o Yes O No
Inclusion and exclusion criteria or boundaries of the model defined (e.g. a CT with 6 mm slice thickness cannot be analyzed)<br><br>o Inclusion or exclusion criteria specified<br>O Inclusion or exclusion criteria not specified
The diversity and distribution across diverse patient groups in the datasets should be reported at training and testing to identify potential biases and apply appropriate corrective measures<br><br>o Mitigation strategies are applied to counter the biases<br>O Mitigation strategies are not applied to counter the biases
Use of post-processing harmonization to reduce multi-center acquisition variability e.g. Combat for HCR and CycleGANs for DL<br><br>o Yes O No
Method and statistical plan pre-registered on a public platform (e.g. www.osf.io)<br><br>O Yes o No

Training dataset coming from one center, two centers, three centers, or more

o One Centre<br>O Two Centres O Three Centres
Multiple segmentations - possible actions are segmentation by different physicians/algorithms/software, perturbing<br>segmentations by (random) noise, segmentation at different breathing cycles. Analyze feature robustness to segmentation variabilities<br><br>o Yes O No
Cut-off analyses - determine risk groups by either median, a previously published cut-off, or report a continuous risk variable or published method. Reduces the risk of reporting overly optimistic results<br><br>o Yes O No
Random permutations to assess the risk of overfitting. Randomize the input variable to get ideally an AUC not different than 0.5 and therefore assess the risk of overfitting.<br><br>o Yes O No
Investigate both handcrafted radiomics and deep learning, or a combination thereof, in an ensemble. Radiomics features may also help in the interpretability of deep learning<br><br>o Comparative analysis or ensemble of HCR and DL approaches<br>O No comparative analysis or ensemble of HCR and DL approaches
Quality Management System<br><br>o Available online with internal audit<br>O ISO certification or equivalent with external audit
Discrimination statistics - report discrimination statistics (e.g., C-statistic, ROC curve, AUC) and their statistical<br>significance (e.g., p-values, confidence intervals). One can also apply a resampling method (for example, bootstrapping, cross-validation).

☑ **A discrimination statistic and its statistical significance are reported** ☑ **A resampling method technique is also applied**
**Calibration statistics - report calibration statistics (e.g., Calibration-in-the-large/slope, calibration plots) and their statistical significance (e.g., p-values, confidence intervals). One can also apply a resampling method (for example, bootstrapping, cross-validation).**

☑ A calibration statistic and its statistical significance are reported ☑ A resampling method technique is also applied
**Comparison with previously published radiomics signatures and models**<br><br>o Yes O No
**Validation - the validation is performed without retraining and adaptation of the cut-off value, providing crucial information about credible clinical performance.**<br><br>O Validation is missing<br>O Validation is based on a dataset from the same institute o Validation is based on a dataset from another institute<br>O Validation is based on two datasets from two distinct institutes O The study validates a previously published signature<br>O Validation is based on three or more datasets from distinct institutes
**Prospective study registered in atrial database (real-world or In Silico), with sample size calculation - provides the highest level of evidence supporting the clinical validity and usefulness of the radiomics biomarker**<br><br>☐ Prospective validation<br>☐ The trial is pre-registered<br>☑ A resampling method technique is also applied

**Algorithm tested in a clinical environment e.g. a department of Radiology or Nuclear medicine**<br><br>**o Yes O No**
**Evaluation should also be carried out concerning the sources of discriminative biases that have been identified**<br><br>**o Yes**

Detect and discuss biological correlates - demonstration of phenotypic differences (possibly associated with underlying gene-protein expression patterns) deepens understanding of radiomics and biology

0 No

o Yes O No

Details on the intrinsic or post-hoc interpretability method or uncertainty estimation method utilized (e.g. attribution maps, SHAP analysis). Evaluation of the explanations using in-silico trials or clinicians.

☑ Details on interpretability methods or uncertainty estimation are available ☑ Sanity and/or evaluation of explanations are available

Comparison to 'gold standard' - assess the extent to which the model agrees with/is superior to the current 'gold standard' method (e.g. Dr. evaluation, TNM-staging for survival prediction, Dr. Assessment). This comparison shows the added value of radiomics

o Yes O No

Potential clinical utility - report on the current and potential application of the model in a clinical setting (e.g., decision curve analysis)

o Yes O No

Cost-effectiveness analysis - report on the cost-effectiveness of the clinical application (e.g., QALYs generated).

O Yes o No

Level of automation for the clinical practice.

At level 0 (No Automation), a clinician performs the clinical task without using the radiomics model.

At level 1 (Clinical Assistance), the clinician uses the radiomics model's prediction for a part of the clinical task.

At level 2 (Partial Automation), the clinician considers the radiomics model's prediction for the clinical task before making the final recommendation.

At level 3 (Conditional Automation), the radiomics model provides the predictions for the clinical task under supervision and the clinician can intervene at anytime.

At level 4 (High Automation), the radiomics model provides the predictions and the clinician's intervention is required for special (out-of-distribution) cases.

At level 5 (Full Automation), the radiomics model provides predictions for the clinical task without human intervention.

**O Level 0 (No Automation)**<br><br>**O Level 1 (Clinical Assistance)**<br><br>**O Level 2 (Partial Automation)**<br><br>**o Level 3 (Conditional Automation) O Level 4 (High Automation)**<br><br>**O Level 5 (Full Automation)**
**The algorithm, source code, and coefficients are made publicly available. Add a table detailing the different versions of software & packages used.**<br><br>**o Yes O No**
**Details on the intrinsic or post-hoc interpretability method or uncertainty estimation method utilized (e.g. attribution maps, SHAP analysis). Evaluation of the explanations using in-silico trials or clinicians.**

☑ Scans are opensource<br><br>☑ The ROI/segmentations are opensource<br><br>☐ Clinical, non-DICOM data, and outcomes are opensource
**Define strategy to update models (frequency, approach, access to data etc)**<br><br>**O Yes o No**

Total score 41 (67.21%)
